# Water‐Soluble Luminescent Trityl Radical for Fluorescence/Magnetic Resonance Dual‐Modal Imaging Probe

**DOI:** 10.1002/advs.77710

**Published:** 2026-09-27

**Authors:** Kosuke Anraku, Satoshi Okada, Akira Sumiyoshi, Kenshiro Matsuda, Kazuhiro Nakamura, Takuya Hosokai, Ken Albrecht

**Affiliations:** ^1^ Interdisciplinary Graduate School of Engineering Sciences Kyushu University Kasuga‐shi Fukuoka Japan; ^2^ Laboratory for Chemistry and Life Science Institute of Integrated Research Institute of Science Tokyo Yokohama‐shi Kanagawa Japan; ^3^ National Institutes for Quantum Science and Technology (QST) Chiba Japan; ^4^ National Institute of Advanced Industrial Science and Technology (AIST) National Metrology Institute of Japan (NMIJ) Ibaraki Japan; ^5^ Institute for Materials Chemistry and Engineering Kyushu University Kasuga‐shi Fukuoka Japan

**Keywords:** chemistry, doublet state, excited state, fluorescence, fluorophore, luminescence, magnetic resonance imaging, photoluminescence, photochemistry, radical

## Abstract

*Tris*(2,4,6‐trichlorophenyl)methyl (TTM) radicals are promising scaffolds for fluorescence imaging (FI) and magnetic resonance imaging (MRI) owing to their long‐wavelength emission and intrinsic paramagnetism. Incorporating donor‐acceptor (D‐A) architectures into TTM radicals has been an established strategy to enhance the brightness and photostability of TTM radicals. However, conventional D‐A‐type TTM radicals exhibit strong charge‐transfer (CT)‐dominated emission that is easily quenched in water due to accelerated non‐radiative decay known as the energy‐gap law. Here, we report water‐soluble TTM radicals (TTM‐*tris*TP3B and TTM‐*tris*TP9B) which exhibit hybridized locally excited and CT character through rational modulation of the donor units and their substitution positions. New radicals exhibited high photostability and deep‐red emission with photoluminescence quantum yields of 1.4% (TTM‐*tris*TP3B, 690 nm) and 1.1% (TTM‐*tris*TP9B, 685 nm) in water. Their dual functionality as both fluorescent probes and MRI contrast agents was demonstrated. TTM‐*tris*TP3B was taken up by living cells, localized near the cell nucleus, and showed fluorescence. The new radicals also provided a clear longitudinal relaxation time (*T*_1_)‐weighted MRI contrast. This work represents the first demonstration of trityl radical functioning as practical FI/MRI dual‐modal imaging probes, establishing a molecular platform without any surfactants or metals in which the fluorophore and electron‐spin source are unified.

## Graphical abstract

Stable water‐soluble luminescent *tris*(2,4,6‐trichlorophenyl)methyl (TTM) radicals, TTM‐*tris*TP3B and TTM‐*tris*TP9B, were designed and successfully synthesized. They exhibited deep‐red emissions and high photostability in water. TTM‐*tris*TP3B exhibited dual functionality as a fluorescence imaging (FI) probe and a magnetic resonance imaging (MRI) contrast agent. This work establishes luminescent radicals as a surfactant‐free and metal‐free molecular platform for FI/MRI dual‐modal imaging probes.

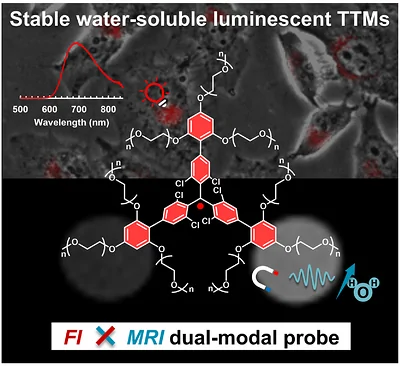

## 1 Introduction

Fluorescence imaging (FI) and magnetic resonance imaging (MRI) are powerful imaging techniques that have played crucial roles in fundamental research and clinical applications [1]. FI has advantages in its high sensitivity, which enables precise tracking of cells and molecular dynamics, while its imaging depth is relatively shallow, ranging from micrometers to centimeters [2]. On the other hand, MRI has advantages, including theoretically unlimited imaging depth, high‐resolution, and high soft‐tissue imaging capabilities, while its sensitivity is relatively low [3, 4]. Therefore, combinations of FI and MRI techniques are anticipated to achieve more reliable diagnostic results. Most conventional FI/MRI dual‐modal probes have consisted of fluorophores and paramagnetic metal complexes covalently linked [5, 6, 7, 8]. Conventional FI/MRI dual‐modal probes have partially independent, electronically distinct molecular structures between the fluorescence probe units and the contrast agent units. These multi‐component strategies are straightforward; however, they complicate the molecular design and obscure the correlation between environmental responses and the resulting signals [5]. Consequently, there is an urgent demand for a single‐component molecular platform where both emissive and spin‐active properties originate from a unified organic framework, ensuring synchronized behavior in complex biological environments.

*Tris*(2,4,6‐trichlorophenyl)methyl (TTM) radical has been known as a representative stable luminescent radical in ambient conditions [9, 10]. TTM shows orange emission with low photoluminescence quantum yield (PLQY) (569 nm, 2% in toluene) stemming from locally excited (LE) states and low photostability (half‐lifetime t_1/2_ is within 1 min in toluene) [11]. On the other hand, a carbazole‐donor substituted donor‐acceptor (D‐A) type TTM, TTM‐Cz, showed largely red‐shifted and brighter emission (671 nm, 60% in toluene) with charge transfer (CT) excited state character and improved photostability (t_1/2_ is more than 1 h in toluene) [11, 12]. D‐A type luminescent radicals show red‐shifted emission originating from the intramolecular CT transition between the highest occupied molecular orbital (HOMO) of the donor and the singly occupied molecular orbital (SOMO) of TTM, and the improvement of PLQY is mainly explained by the enhancement of the transition dipole moment and the oscillator strength [10]. The drastic improvement in the photostability of TTM‐Cz arises from the stabilization of the excited state compared to bare TTM, resulting in an increase in the activation energy for the photochemical degradation reaction [13, 14, 15, 16]. The D‐A architecture is a reasonable strategy for luminescent radicals to achieve long‐wavelength emission, bright luminescence, and high stability [10, 17, 18, 19, 20, 21, 22].

TTM radical derivatives are considered metal‐free FI probes in biological environments due to their red‐to‐near‐infrared emission [23, 24, 25, 26, 27, 28, 29, 30, 31]. Water‐soluble LE‐type TTM radicals have been reported, but their photophysical properties are poor, as mentioned above [24, 25, 32]. On the other hand, utilization of D‐A type TTMs in aqueous biological environments remains a major challenge because D‐A emitters are typically quenched in highly polar water due to the excessive red‐shift in PL, known as the energy gap law [33, 34]. Fabrication of nanoparticles (NPs) by incorporating hydrophobic D‐A [26, 27, 28, 29] or LE [30] type radicals into hydrophobic hosts, followed by surfactant‐assisted solubilization, has been reported by a few groups. This is a reliable strategy to keep the environmental polarity around the radicals low and extract high luminescence performance. However, this NP approach limits access of water to the radical species, thereby putting their use as MRI contrast agents at a disadvantage. Intrinsically water‐soluble luminescent radicals that retain good photophysical properties are eagerly anticipated.

Stable luminescent radicals are potential candidates for a new class of metal‐free FI/MRI dual‐modal probes, owing to their inherently unified electron‐spin‐fluorophore structure. Currently, only one D‐A type water‐soluble luminescent radical has been reported as a FI/MRI dual‐modal probe; however, its PLQY (777 nm, 0.006%) and longitudinal relaxivity (0.02 mm ^−1^ s^−1^) are relatively low. The typical D‐A structure incorporating a strong donor unit results in a red‐shift of the emission and accelerates non‐radiative decay. This structure also induces concentration quenching due to the amphiphilic structure, leading to aggregates and weakening the interaction between an electron spin and water protons [34]. Properly designed water‐soluble luminescent radicals will fulfill the requirements of not only FI probes, but also MRI contrast agents because they have an electron spin that can interact with a proton of water [35]. Luminescent radicals (spin quantum number *S* = 1/2) can serve as biocompatible alternatives to metal‐based MRI contrast agents, such as Gd(III) complexes, which have been associated with side effects including nephrogenic systemic fibrosis and Gd accumulation in the brain.

Herein, we designed tri‐poly(ethylene glycol) (PEG)‐functionalized benzene‐substituted water‐soluble luminescent radicals, TTM‐*tris*TP3B and TTM‐*tris*TP9B, and successfully demonstrated their use in FI/MRI applications. To achieve stable fluorescence in aqueous environments, the molecular design was guided by two key considerations. (i) Employment of a D‐A architecture incorporating a relatively weak electron‐donating group enabling moderate excited‐state CT character [36, 37, 38]. (ii) Donor substituents were introduced to the TTM core with the three *para*‐positions in a manner that covers and encapsulates the hydrophobic TTM core with PEG chains. This donor‐trisubstituted structure was expected to reduce the permanent dipole moment and aggregation. (i) and (ii) strategies allow TTMs to possess a hybridized locally excited and charge‐transfer (HLCT) character, resulting in maintained emissive properties in water [39]. TTM‐*tris*TP3B and TTM‐*tris*TP9B exhibited deep‐red emissions with high photostability and moderate PLQY in water among the reported water‐soluble luminescent radicals. TTM‐*tris*TP3B and TTM‐*tris*TP9B were tested for FI and MRI. TTM‐*tris*TP3B exhibited good performance as both an FI probe and an MRI contrast agent. This work represents the first demonstration of trityl radicals functioning as FI/MRI dual‐modal imaging probes, establishing a surfactant‐free and metal‐free molecular platform in which both fluorescence and electron‐spin activity originate from a unified molecular framework. Unlike conventional dual‐modal probes based on multiple functional components, this design intrinsically integrates emissive and spin‐active properties within a single structure. This unified architecture allows a single molecular response to be probed through two independent physical readouts, enabling intrinsic cross‐validation between fluorescence and magnetic resonance signals and reducing ambiguity in their interpretation.

## 2 Results and Discussion

### 2.1 Synthesis and Characterization

New water‐soluble luminescent radicals, TTM‐*tris*TP3B and TTM‐*tris*TP9B, were synthesized via Suzuki‐Miyaura cross‐coupling, Williamson ether reaction, and radicalization (Scheme 1). Synthesis of water‐soluble luminescent radicals was started from Suzuki‐Miyaura cross‐coupling between TTM radical precursor (HTTM) and pinacol boronated ester of 1,3,5‐trimethoxybenzene to obtain the tri(1,3,5‐trimethoxybenzene)‐substituted HTTM (HTTM‐*tris*TMB). HTTM‐*tris*TMB was treated with BBr3 to convert the methoxy groups to hydroxy groups, and then reacted with tosylated polyethylene glycol to obtain HTTM‐trisTP3B and HTTM‐trisTP9B, which have different PEG chain lengths. HTTM‐*tris*TP3B and HTTM‐*tris*TP9B were treated with tetrabutylanmonium hydroxide (TBAOH) and subsequently one‐electron oxidized by *p*‐chloranil to obtain the radical compounds, TTM‐*tris*TP3B and TTM‐*tris*TP9B. HTTM‐*tris*TP3B and HTTM‐*tris*TP9B were characterized by ^1^H NMR (nuclear magnetic resonance), ^13^C NMR, MALDI TOF MS, and elemental analysis. TTM‐*tris*TP3B and TTM‐*tris*TP9B were characterized by MALDI TOF MS, elemental analysis, and electron‐spin resonance (ESR) spectroscopy. The radical purities of TTM‐*tris*TP3B and TTM‐*tris*TP9B were calculated to be 63% and 53%, respectively, by comparing the signal strength with that of the standard reference (TEMPOL) in ESR spectroscopy (Figure S11 and Table S1). The impurities were confirmed by ^1^H NMR to be the radical precursors that cannot be separated due to their structural similarities (Figures S9 and S10). PEGylated TTMs, TTM‐*tris*TP3B and TTM‐*tris*TP9B, were successfully synthesized and characterized.

**SCHEME 1:**
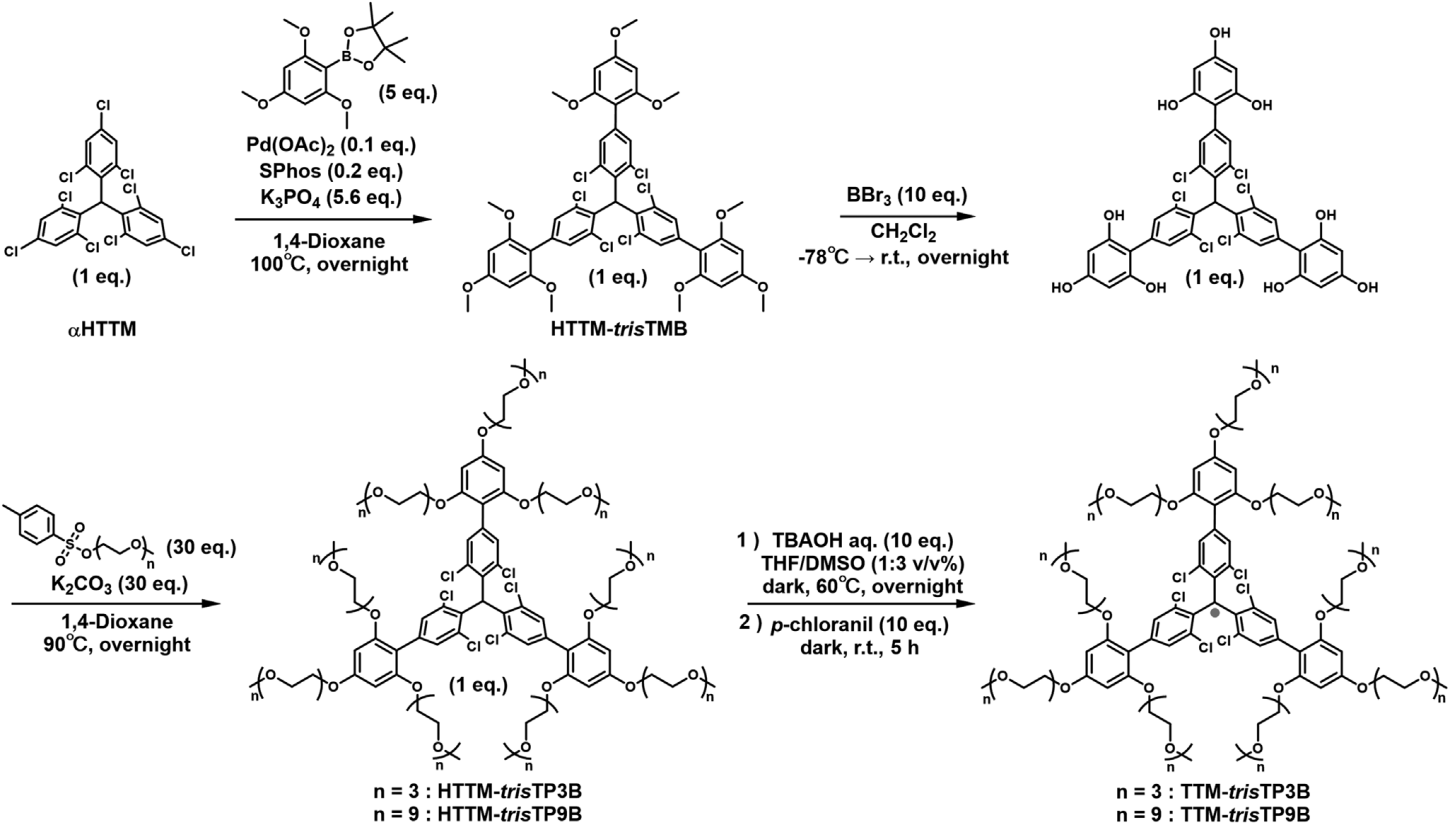
Synthesis of TTM‐*tris*TP3B and TTM‐*tris*TP9B.

### 2.2 Optical Properties in Organic Solvents

UV–vis absorption and PL spectra of TTM‐*tris*TP3B and TTM‐*tris*TP9B in toluene, THF, chloroform, dichloromethane, and methanol were measured (Figure 1 and Table 1 and Figure S2). Both TTM‐*tris*TP3B and TTM‐*tris*TP9B exhibited three characteristic absorption bands at around 390, 420, and 580 nm. The bands were assigned using time‐dependent density functional theory (TD‐DFT) calculations and natural transition orbitals (NTOs) analysis at UPBE0/6‐31+G(d,p) level (Figure 3). The strong absorption bands observed at approximately 390 and 420 nm were primarily attributed to the LE transitions localized on the TTM moiety and conjugated TTM‐trialkoxybenzene unit, respectively, while the weak and broad absorption band observed at approximately 580 nm was mainly attributed to the HLCT transition from the whole molecule to the TTM acceptor. In toluene, THF, chloroform, and dichloromethane, both TTM‐*tris*TP3B and TTM‐*tris*TP9B exhibited bright red emission at 646–655 nm and PLQYs of 46%–50% for TTM‐*tris*TP3B and 650–661 nm and 41%–48% for TTM‐*tris*TP9B (Figure 1 and Table 1 and Figure S2). Note that the precursors do not have absorption around 405 and 416 nm (excitation wavelengths for PL lifetime, PL spectra, and PLQY measurements), and only the radicals are excited for these measurements (Figure S12). Differing from conventional D‐A radicals, both TTM‐*tris*TP3B and TTM‐*tris*TP9B exhibited only a slight red‐shift in their PL spectra, maintaining the high PLQY, despite a significant increase in solvent polarity from toluene to methanol. This observation suggests the potential contribution of HLCT character to their emissive states [25, 34]. Surprisingly, both TTM‐*tris*TP3B and TTM‐*tris*TP9B exhibited relatively high PLQY of 16% even in a highly polar solvent, methanol. TTM‐*tris*TP3B and TTM‐*tris*TP9B showed small solvent‐dependent PL, and this will be an advantage for use in highly polar solvents compared to conventional luminescent radicals.

**FIGURE 1:**
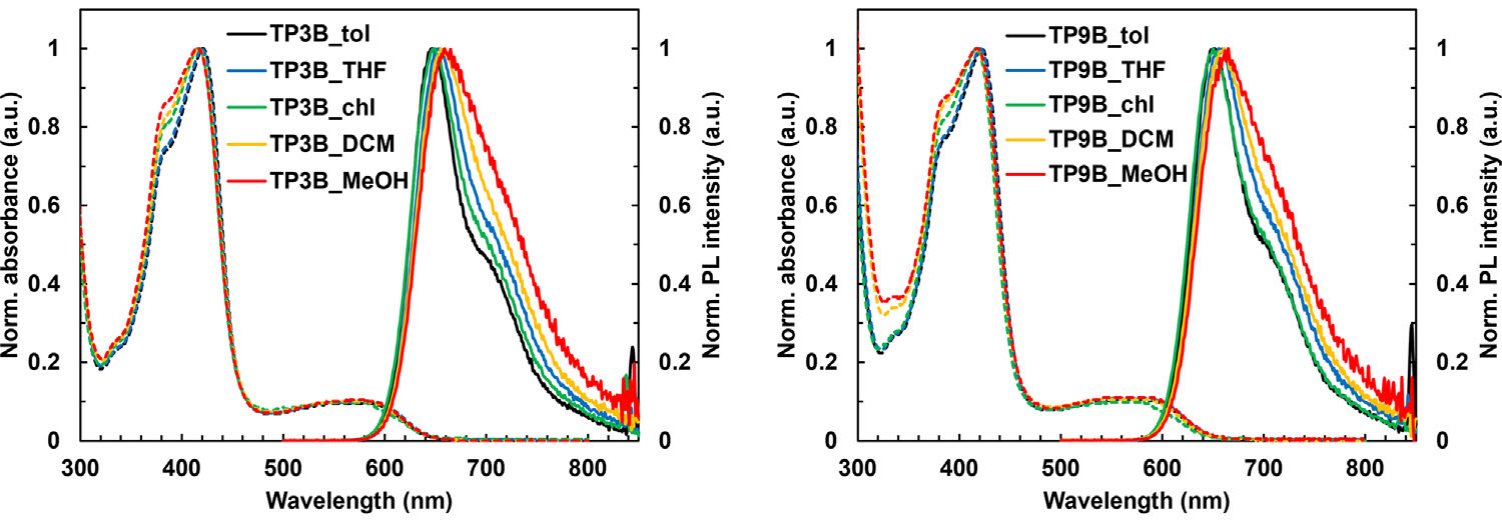
UV–vis absorption (broken line) and PL (solid line) spectra of (left) TTM‐*tris*TP3B (TP3B) and (right) TTM‐*tris*TP9B (TP9B) in toluene (tol), tetrahydrofuran (THF), chloroform (chl), dichloromethane (DCM), and methanol (MeOH) (10^−5^ M).

**TABLE 1:** Photophysical properties of TTM‐*tris*TP3B and TTM‐*tris*TP9B in toluene and water.

	Solvent (*E*_T_(30))^a^	λ_abs_ (nm)^b^	λ_em_ (nm)^b^	PLQY (%)^b^	τ(ns)^b^	*k*_r_ (s^−1^)^c^	*k*_nr_ (s^−1^)^c^
TTM‐*tris*TP3B	Toluene (33.9)	572	646	47	30	1.6 × 10^7^	1.8 × 10^7^
	Water (63.1)	571	690	1.4	1.5	9.3 × 10^6^	6.6 × 10^8^
TTM‐*tris*TP9B	Toluene (33.9)	560	651	48	30	1.6 × 10^7^	1.7 × 10^7^
	Water (63.1)	564	685	1.1	0.9	1.2 × 10^7^	1.1 × 10^9^

^a^ Solvent polarity parameters cited from ref. [42].

^b^ λ_abs_: absorption, λ_em_: PL peak wavelength, PLQY: photoluminescence quantum yield, and PL lifetime measured in toluene and water (10^−5^ M).

^c^ *k*_r_: radiative and *k*_nr_: non‐radiative decay rate constant.

### 2.3 Optical Properties in Water

UV–vis absorption and PL spectra of TTM‐*tris*TP3B and TTM‐*tris*TP9B were measured in water (Figure 2 and Table 1). TTM‐*tris*TP3B and TTM‐*tris*TP9B exhibited three characteristic absorption bands at around 390, 420, and 580 nm without any light scattering, similar to those observed in organic solvents. This result suggested that TTM‐*tris*TP3B and TTM‐*tris*TP9B are water‐soluble radicals. Dynamic light scattering experiments of HTTM‐*tris*TP3B and HTTM‐*tris*TP9B revealed that no distinct aggregates were found at a concentration of 10^−5^ M (the concentration used for UV–vis and PL measurements), but aggregates with a hydrodynamic diameter of around 100–200 nm were detected at concentrations of 10^−4^ and 10^−3^ M (Figure S26). Note that the measurements were performed using the closed‐shell radical precursors to avoid the influence of radical emission on the experimental results. The molar absorption coefficients (ε) in water, corrected by the radical purities, were determined to be 3.0 × 10^4^/3.5 × 10^3^ M^−1^ cm^−1^ (at 413/571 nm) for TTM‐*tris*TP3B and 3.2 × 10^4^/4.2 × 10^3^ M^−1^ cm^−1^ (at 416/564 nm) for TTM‐*tris*TP9B, respectively. TTM‐*tris*TP3B and TTM‐*tris*TP9B exhibited approximately 35 nm red‐shifted PL compared to the PL in toluene, with over 1% of PLQYs (690 nm/1.4% for TTM‐*tris*TP3B and 685 nm 1.1% for TTM‐*tris*TP9B). The PL spectra of TTM‐*tris*TP3B and TTM‐*tris*TP9B in toluene exhibited a shoulder at approximately 700 nm, whereas those in water were broad and lacked vibronic structures (Figures 1 and 2). These observations suggested that the excited states of TTM‐*tris*TP3B and TTM‐*tris*TP9B were stabilized slightly with the increase of the solvent polarity. The PLQY of both TTM‐*tris*TP3B and TTM‐*tris*TP9B in water significantly decreased compared to those in toluene, from approximately 50% to 1%. The drops in PLQY of radicals were also observed in protic solvent, methanol (PLQY was 16% for both radicals). One of the reasons for the significant drops in PLQY was possibly attributed to the increased proximity of protic solvent molecules to the radical chromophore induced by the PEG chains. Vibronic coupling between the excited state and the O‐H stretching modes of protic solvent molecules might enhance non‐radiative decay pathways, leading to fluorescence quenching [40, 41]. To confirm the validity of this consideration, PLQYs of radicals in deuterated water (D_2_O) were measured (Figure S13 and Table S2). The vibronic energy of the O‐D stretching mode is lower than that of the O–H stretching mode because deuterium is heavier than hydrogen. Based on the energy gap law, vibronic coupling between the excited state and the O‐D stretching mode of D_2_O is less efficient compared to the O–H stretching mode of H_2_O. In other words, the PLQY of radicals in D_2_O is expected to be improved compared to that in H_2_O. Indeed, an increase in PLQYs in D_2_O was observed for both TTM‐*tris*TP3B (3.1%) and TTM‐*tris*TP9B (3.6%) without any spectral changes (Figure S13 and Table S2). Furthermore, in comparison to calculated radiative (*k*_r_) and non‐radiative decay rate constants (*k*_nr_) in D_2_O with those in H_2_O, *k*_r_ values in D_2_O (9.4 × 10^6^ s^−1^ for TTM‐*tris*TP3B, and 1.8 × 10^7^ s^−1^ for TTM‐*tris*TP9B) were almost same with those in H_2_O (9.3 × 10^6^ s^−1^ for TTM‐*tris*TP3B, and 1.2 × 10^7^ s^−1^ for TTM‐*tris*TP9B), however, *k*_nr_ values in D_2_O (2.9 × 10^8^ s^−1^ for TTM‐*tris*TP3B, and 4.8 × 10^8^ s^−1^ for TTM‐*tris*TP9B) were suppressed to be approximately one‐half compared to those in H_2_O (6.6 × 10^8^ s^−1^ for TTM‐*tris*TP3B, and 1.1 × 10^9^ s^−1^ for TTM‐*tris*TP9B) (Table S2). These results support the assumption that protic O–H stretching increases the non‐radiative decay pathway and diminishes PLQYs in water. TTM‐*tris*TP3B and TTM‐*tris*TP9B showed water solubility and deep‐red emission in water, and they can be potential candidates for fluorescence imaging probes.

**FIGURE 2:**
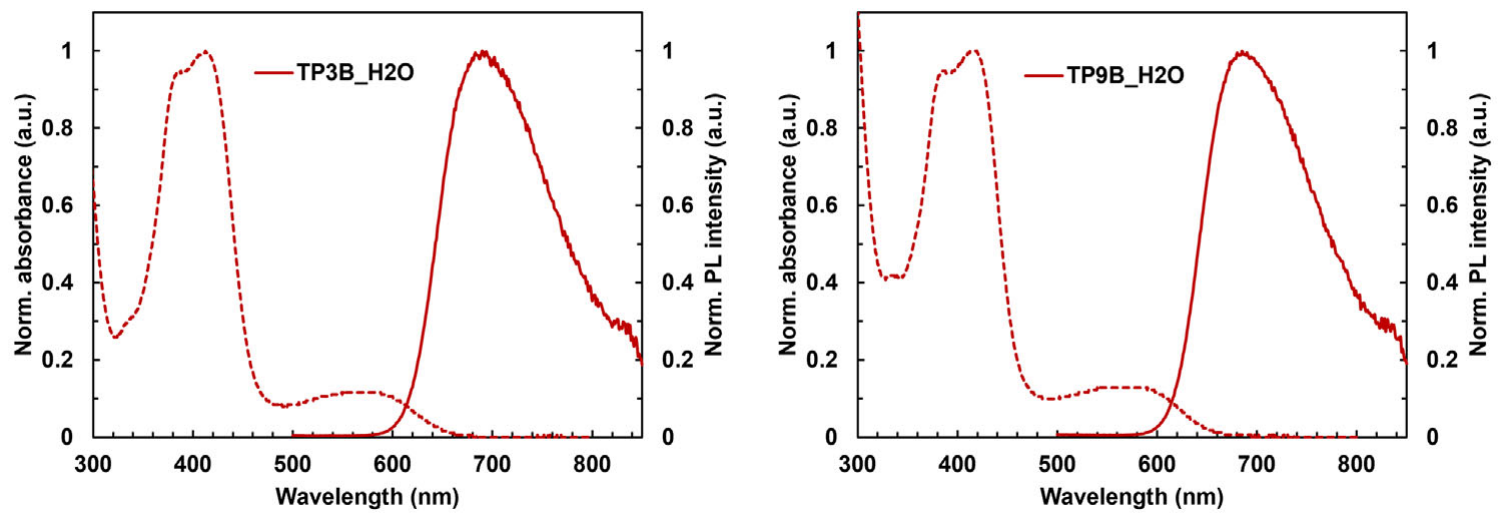
UV–vis absorption (broken line) and PL (solid line) spectra of (left) TTM‐*tris*TP3B (TP3B) and (right) TTM‐*tris*TP9B (TP9B) in water (H2O) (10^−5^ M).

### 2.4 Theoretical Calculations

To understand the electronic structures of TTM‐*tris*TP3B and TTM‐*tris*TP9B, DFT calculations using Gaussian 16 were conducted [43]. Note that the calculations were conducted for a methoxylated radical due to the conformational complexity of PEG chains. The structural optimization in the ground state was performed at UB3LYP/6‐31G(d,p) level in vacuum, and the TD‐DFT calculations were performed at UPBE0/6‐31+G(d,p) level in toluene (Figure 3 and Figure S16). According to the visualized distributions of molecular orbitals in the optimized structure, αHOMO (245α), αLUMO (246α), and βHOMO (244β) are delocalized over the whole molecule, while βLUMO (245β) is localized on the TTM moiety. The absorption spectrum obtained by the TD‐DFT calculation exhibited good agreement with the trend in the line shape of the experimental absorption spectrum (Figure S17). D_1_ ← D_0_, D_7_ ← D_0_, and D_8_← D_0_ transitions correspond to the absorption band at approximately 580, 420, and 390 nm in experimental absorption spectra, respectively (Figure S17). The NTOs of the methoxylated radical were calculated and visualized. From the NTO analysis, the D_7_ ← D_0_ and D_8_ ← D_0_ transitions were found to involve multiple NTO pairs, but they were primarily characterized by the LE transitions of TTM and the conjugated TTM‐trimethoxybenzene unit (around 50% of contributions), respectively (Figure S18). For the D_1_ ← D_0_ transition, the hole in the 1st NTO pair was delocalized over the whole radical; however, the particle in the 1st NTO pair was localized on the TTM moiety. This result suggests that the D_1_ ← D_0_ transition does not have a clear CT character; in other words, the D_1_ ← D_0_ transition has an HLCT character. The hole‐electron analysis for the D_1_ state was performed utilizing Multiwfn 2026.7.15 (Figure S19) [44, 45]. The hole density was delocalized over the whole molecule, while the electron density was predominantly localized on the TTM moiety. The simulated spatial overlap index (S_r_) and centroid distance between hole and electron (D) were 0.55551 and 2.161 Å, respectively. Comparing these values to those of a typical D‐A type TTM radical, TTM‐Cz (S_r_ = 0.3900 and D = 5.879 Å), the D_1_ state of the water‐soluble radicals possesses weaker CT nature [46]. The S_r_ value of our radical is within a range defined as HLCT, 0.4–0.75, which supports the fact that its D_1_ state is an HLCT state [47, 48, 49]. The DFT calculation results reveal that the excited state of the new water‐soluble radicals has an HLCT nature and explain the low sensitivity to solvent polarity of the new radicals.

**FIGURE 3:**
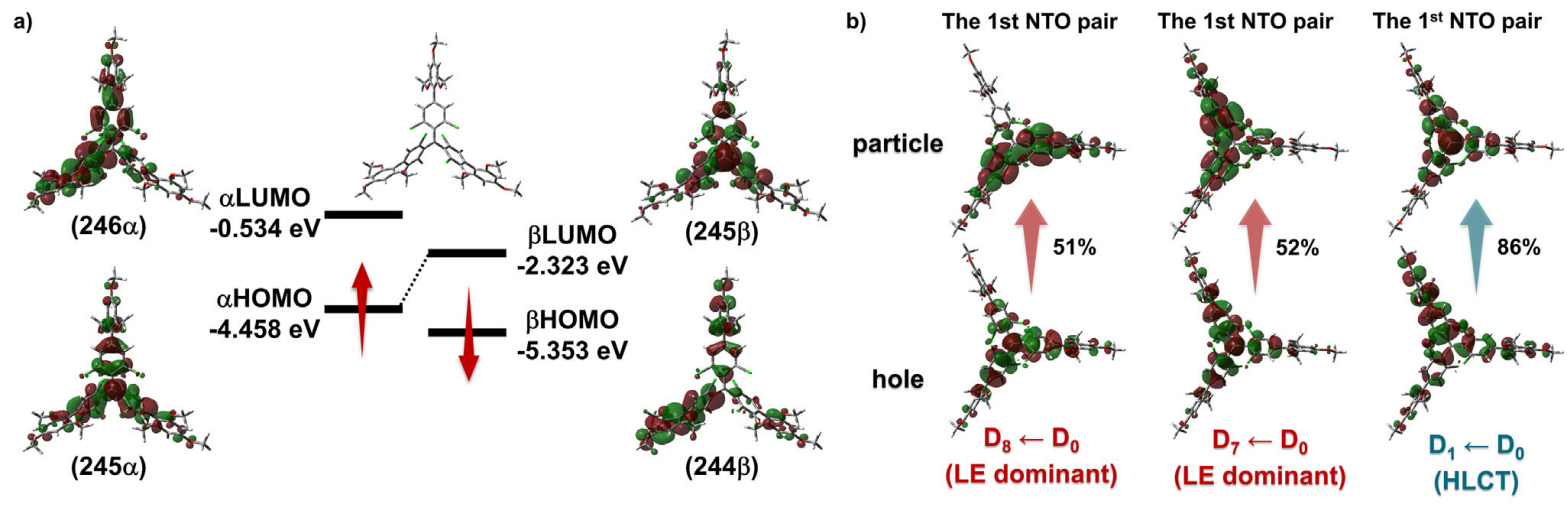
a) Energy levels and spatial distributions of the frontier molecular orbitals of the methoxylated radical obtained from DFT calculations at the UB3LYP/6‐31G(d,p) level. b) NTOs for the D_1_ ← D_0_, D_7_ ← D_0_, and D_8_← D_0_ transitions of methoxylated radical calculated using TD‐DFT at the UPBE0/6‐31+G(d,p) level in toluene. The hole and particle orbitals are shown at the top and bottom, respectively. The 1st NTO pairs correspond to the dominant transition.

### 2.5 Photostability

The photostability of TTM‐*tris*TP3B and TTM‐*tris*TP9B was investigated by monitoring the PL peak intensity during continuous irradiation with a 355 nm pulsed laser vs. the laser irradiation times in toluene and water (Figure 4). The absorbance of radical solutions was adjusted to 0.5 at 355 nm, and the solutions were stirred during the measurement using a homemade apparatus [50]. The half‐lifetimes (t_1/2_) of radicals were calculated by using the following Equation (1). In the photostability experiments, approximately 99% of the photons were estimated to be absorbed by the radicals based on the ratio of the ε values of the radicals and the precursor impurity at the excitation wavelength. In addition, the monitoring PL wavelength for these experiments was around 690 nm, where no PL of the precursor exists (Figure S12). Both TTM‐*tris*TP3B and TTM‐*tris*TP9B in toluene exhibited significantly improved t_1/2_ (1.6 × 10^4^ s) compared to the original TTM (33 s) [34]. This significant improvement in photostability might be due to an increase in the activation energy for the photochemical degradation reaction compared to the original TTM radical, induced by the introduction of the trialkoxybenzene donor and relatively short excited lifetime [14, 15, 16]. Furthermore, in water, the PL intensity of TTM‐*tris*TP3B and TTM‐*tris*TP9B did not decrease, but rather a slight increase during continuous laser irradiation was observed (Figure S20). This phenomenon was observed without any spectral change in the PL profiles, indicating the high photostability of these radicals (Figure S20).

**FIGURE 4:**
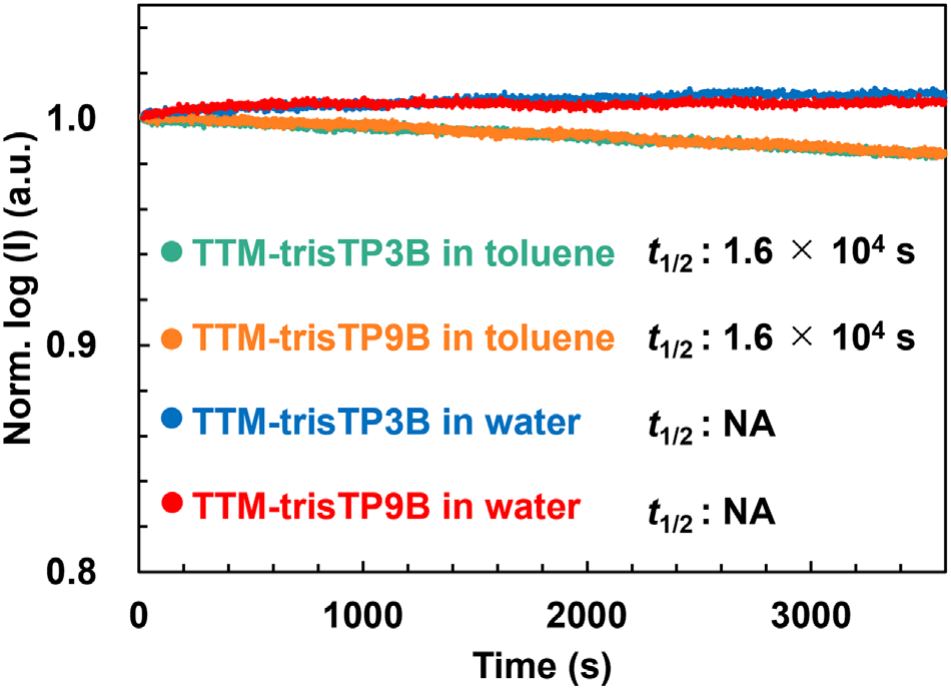
Time‐dependent PL peak intensity (*l*) of TTM‐*tris*TP3B and TTM‐*tris*TP9B under continuous irradiation with a 355 nm pulsed laser irradiation in toluene and water (power: 2.0 mW, beam diameter (Gaussian beam, 1/e^2^ level, FWHM): ∼3 mm, pulse width: 28 ps, repetition rate: 10 Hz). The inset shows the corresponding half‐lifetimes (t_1/2_).

Additional control experiments were conducted to clarify the origin of the increase in PL intensity observed during the photostability measurement of TTM‐*tris*TP3B in water (Figures S21 and S22). First, the effect of laser irradiation was evaluated by comparing two irradiation conditions: continuous irradiation and intermittent irradiation applied only once every 5 min. A similar increase in PL intensity was observed under both conditions, indicating that the continuous laser irradiation is not responsible for the enhancement of PL. In contrast, when the solution was not stirred, no appreciable increase in PL intensity was detected (Figure S21). These results indicate that the increase in PL intensity is induced by stirring rather than by laser irradiation. Furthermore, the increased PL intensity observed after photostability measurements did not return to its initial value even after the sample was kept in the dark for 1 h without laser irradiation. This finding suggests that the radical‐water system does not simply revert to its initial state once the PL enhancement has occurred. Notably, the increase in PL intensity was accompanied by a slight decrease in the intensity of laser scattering monitored at 355 nm (Figure S22). Taken together, these observations suggest that the increase in PL intensity may be associated with the partial disaggregation of radical aggregates induced by stirring (Figure S26), and that this phenomenon is irreversible or only slowly recovers over the time scale performed in this study. In any case, these water‐soluble radicals are significantly stable in water. The absence of photodegradation clearly distinguishes our radicals from some clinically used fluorescent probes, such as indocyanine green (ICG), which suffer from rapid photodegradation [51].

(1) $$I\left(t\right)=I_{0}{\left(\frac{1}{2}\right)}^{\frac{t}{t_{1/2}}}$$

### 2.6 Fluorescence Imaging

To confirm the applicability of TTM‐*tris*TP3B and TTM‐*tris*TP9B as FI probes, FI experiments were conducted in cells at a final concentration of 10 µm (Figure 5 and Figure S23). TTM‐*tris*TP3B was taken up by HeLa cells within 1 h, whereas TTM‐*tris*TP9B showed negligible cellular uptake, presumably due to the longer PEG chains imparting enhanced stealth properties. The fluorescence signals of TTM‐*tris*TP3B were predominantly localized in the perinuclear region and persisted for at least 24 h. The mechanism of the radical localization in the perinuclear region has not been fully understood, but elucidating this mechanism could facilitate the development of radical‐based FI probes that target specific organelles. The persistent fluorescence of TTM‐*tris*TP3B in the presence of endogenous reductants could be attributed to the significantly lower reduction potential of TTM radical derivatives (around −0.4 V vs. SHE (standard hydrogen electrode)) [34] compared to endogenous reductants (around −0.23 V vs. SHE) [52] and the significant steric hindrance provided by the PEG chains protecting the radical center. To evaluate the cytotoxicity of TTM‐*tris*TP3B, an MTT assay (MTT: 3‐(4,5‐Dimethylthiazol‐2‐yl)‐2,5‐diphenyltetrazolium bromide) was performed on HeLa Cells for 48 h incubation (Figure S24). The IC_50_ (50% inhibitory concentration) value was determined to be 13 µm, indicating that TTM‐trisTP3B exhibits concentration‐dependent cytotoxicity upon prolonged exposure. Based on these results, TTM‐*tris*TP3B can be used as an FI probe under the conditions examined, although its cytotoxicity at high concentrations and prolonged exposure should be considered.

**FIGURE 5:**
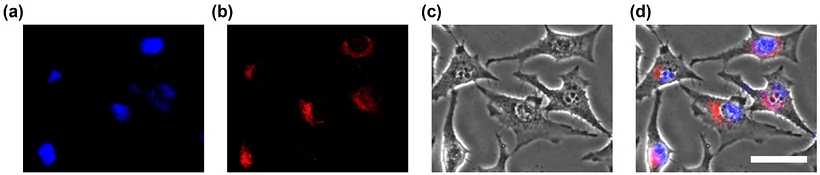
Fluorescence imaging of HeLa cells incubated with 10 µm of TTM‐*tris*TP3B for 24 h. (a) 4′,6‐diamidino‐2‐phenylindole (DAPI), (b) TTM‐*tris*TP3B, (c) phase contrast, and (d) merged images of (a–c). Scale bar = 50 µM. The nuclear region was visualized using DAPI.

### 2.7 Magnetic Resonance Imaging

To investigate the applicability of TTM‐*tris*TP3B and TTM‐*tris*TP9B as MRI contrast agents, the longitudinal relaxation time (*T*_1_) of protons of water was measured, and *T*_1_‐weighted phantom images were obtained (Figure 6). In MRI, proton nuclei of water are observed, and differences in their relaxation times provide contrast in the images. The relaxation time of proton nuclei around paramagnetic materials is shortened by the magnetic interaction between a proton nucleus and an unpaired electron spin (so‐called paramagnetic relaxation enhancement (PRE) effect). The *T*_1_ values were measured using a 1 T NMR magnet at different concentrations of radical aqueous solutions to determine the longitudinal relaxivity (*r*_1_), which reflects the ability of the radicals as MRI contrast agents. Note that the concentrations of originally prepared radical aqueous solutions were multiplied by the radical purity and plotted to estimate true *r*_1_ values. The inverse of the *T*_1_ values linearly increased with increasing radical concentration, indicating that *T*_1_ values were shortened due to the PRE effect. The *r*_1_ values for TTM‐trisTP3B and TTM‐trisTP9B were calculated to be 0.049 and 0.089 mm ^−1^ s^−1^, respectively, using Equation (2). These values were approximately 2.5‐ and 4.5‐times larger than the *r*_1_ values of TTM‐PEG3 (0.021 mm ^−1^ s^−1^), a previously reported water‐soluble radical [34]. The reason for the low *r*_1_ values, which remain low compared to the clinically used Gd (III) complexes, was primarily due to the intrinsically smaller spin quantum number (S = 1/2) than Gd complexes (S = 7/2). A further reason might be the formation of some aggregates, which limits the interaction between electron spins and water protons (Figure S26) [34]. To confirm the utility of TTM‐*tris*TP3B and TTM‐*tris*TP9B as MRI contrast agents, *T*_1_‐weighted MRI phantom images were measured. Note that the radical concentrations were calibrated by radical purity in the same way as the plots of the *T*_1_^−1^ vs. radical concentrations. *T*_1_‐weighted MRI phantom images provided a clear enhancement of the imaging contrast with increasing radical concentrations, especially in TTM‐*tris*TP9B. This result suggested that water‐soluble carbon center radicals are potential candidates for MRI contrast agents.

(2) $$T_{1}^{-1}=r_{1}C+T_{0}^{-1}$$

**FIGURE 6:**
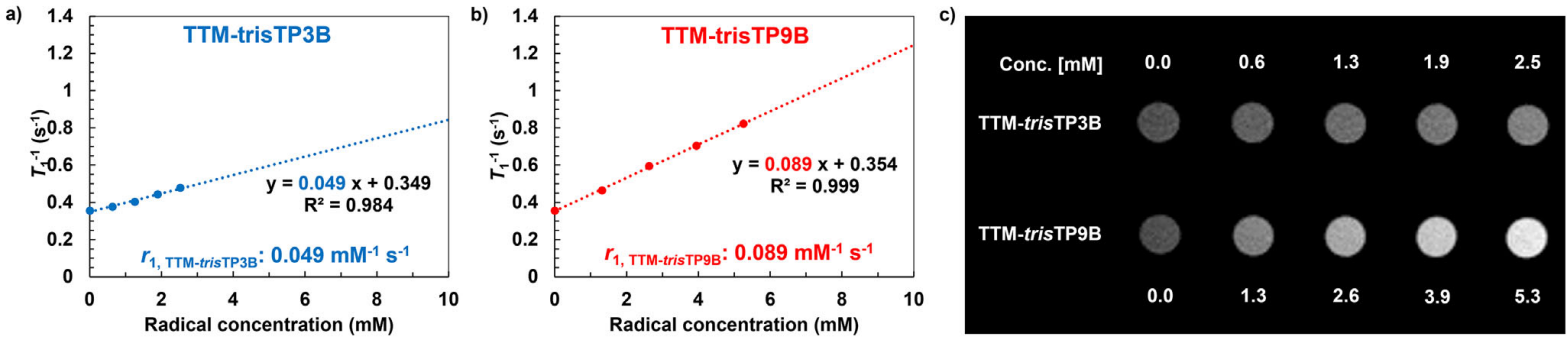
Plot of *T*_1_ relaxation rates of H_2_O protons vs. the concentration of (a) TTM‐*tris*TP3B and (b) TTM‐*tris*TP9B in water under 1 T of magnetic field. (c) *T*_1_‐weighted MRI phantom images at each concentration of radical aqueous solutions under 1 T of magnetic field.

## 3 Conclusion

In summary, D‐A type water‐soluble TTM radicals, TTM‐*tris*TP3B and TTM‐*tris*TP9B, were successfully developed. Their highly symmetric molecular structures, incorporating relatively weak donor units (tri‐PEGylated benzene), enabled solvent‐polarity‐insensitive PL with a HLCT character. TTM‐*tris*TP3B and TTM‐*tris*TP9B exhibited high PLQY in organic solvents while allowing the radicals to retain luminescence in water. These radicals exhibited deep‐red emissions in water and high photostability. The high photostability of these radicals stems from the CT excited state induced by the D‐A structure, unlike the conventional LE‐type water‐soluble radicals reported to date. TTM‐*tris*TP3B showed dual functionality as a fluorescent probe and an MRI contrast agent. This work establishes luminescent trityl radicals as a surfactant‐free and metal‐free molecular platform for FI/MRI dual‐modal imaging probes and provides a useful design strategy for developing water‐compatible luminescent radical probes for bioimaging applications.

## 4 Experimental

### 4.1 Statistical Analysis

Statistical hypothesis testing was not performed in this study. Cell viability data are presented as mean ± SD, with the sample size (n) indicated in the corresponding figure legend in Supporting Information; *n* = 3 represents three replicates.

## Author Contributions

K. Anraku, K. Albrecht and T.H. contributed to the acquisition of the funds for this project. The overall design of the investigation was done by K. Anraku with assistance from K. Albrecht and T.H. Most synthesis and measurements of compounds were performed by K. Anraku, with the assistance of K.M. and K.N. Theoretical calculations were performed by K. Anraku. FI experiments and the analysis were undertaken by S.O. MRI experiments and the analysis were undertaken by A.S., with the assistance of S.O. Major contributions to data analysis and curation were carried out by K. Anraku, T.H., and K. Albrecht with smaller contributions from all others. The original draft of the paper was prepared by K. Anraku and revised based on comments from K. Albrecht, T.H., and S.O. All authors have given approval to the final version of the manuscript.

## Conflicts of Interest

The authors declare no conflicts of interest.

## Supporting information




**Supporting File**: advs77710‐sup‐0001‐SuppMat.pdf.

## Data Availability

The data that support the findings of this study are available from the corresponding authors upon reasonable request.
