# Supplementary material for: Water‐Soluble Luminescent Trityl Radical for Fluorescence/Magnetic Resonance Dual‐Modal Imaging Probe
Source: Adv Sci (Weinh). 2026 Sep 27:e77710. Online ahead of print. doi: 10.1002/advs.77710 (PMC13617257; doi:10.1002/advs.77710)
Supplement: Supplementary file 1 [file ADVS-9999-e77710-s001.pdf]

Supporting Information

Water-Soluble Luminescent Trityl Radical for Fluorescence/Magnetic Resonance Dual-Modal Imaging Probe

Kosuke Anraku,[a] Satoshi Okada*,[b] Akira Sumiyoshi,[c] Kenshiro Matsuda,[a] Kazuhiro Nakamura,[a] Takuya Hosokai*,[d] and Ken Albrecht*[e]

[a] Kosuke Anraku, Kenshiro Matsuda, Kazuhiro Nakamura

Interdisciplinary Graduate School of Engineering Sciences, Kyushu University

6-1 Kasuga-Koen, Kasuga-shi, Fukuoka 816-8580, Japan.

[b] Satoshi Okada

Laboratory for Chemistry and Life Science, Institute of Integrated Research, Institute of Science Tokyo

4259 Nagatsuta, Midori, Yokohama-shi, Kanagawa 226-8501, Japan.

E-mail: okada.s.e21a@m.isct.ac.jp

[c] Akira Sumiyoshi

National Institutes for Quantum Science and Technology (QST)

4-9-1 Anagawa, Inage-ku, Chiba-shi, Chiba 263-8555, Japan

[d] Takuya Hosokai

National Institute of Advanced Industrial Science and Technology (AIST)

National Metrology Institute of Japan (NMIJ)

Tsukuba Central 5, 1-1-1 Higashi, Tsukuba-shi, Ibaraki 305-8565, Japan.

E-mail: t.hosokai@aist.go.jp

[e] Ken Albrecht

Institute for Materials Chemistry and Engineering, Kyushu University

6-1 Kasuga-Koen, Kasuga-shi, Fukuoka 816-8580, Japan.

E-mail: albrecht@cm.kyushu-u.ac.jp

Table of Contents

Experimental section S3
Synthesis S5
NMR spectra S13
ESR spectra S19
UV-vis absorption and PL spectra S20
PL lifetime S21
DFT calculations S23
Photostability S26
Fluorescence imaging S28
MRI experiments S29
DLS measurements S31

Experimental section

Chemicals: All chemicals were purchased from FUJIFILM Wako Pure Chemical Co., TCI chemicals, Aldrich, or BLD pharm and used without further purification unless otherwise noted. Dehydrated solvents were used in all syntheses, and spectroanalytical grade solvents were used in all spectroscopic measurements. The MilliQ-water was used in all spectroscopic measurements in water.

Cells: HeLa cells (TKG 0331, RRID: CVCL_0030) were obtained from the Cell Resource Center for Biomedical Research, Institute of Development, Aging and Cancer, Tohoku University, Japan. Cells were cultured in Dulbecco's modified Eagle's medium (DMEM) supplemented with 10% fetal bovine serum and 1% penicillin-streptomycin at 37 °C under 5% CO_2.

General: The NMR spectra were obtained using JNM-ECZ400 (400MHz) and JNM-ECA600 (600MHz) (JEOL). ^{1}H NMR and ^{13}C NMR spectra were measured with TMS as the internal standard. The elemental analysis was performed at the Service Center of the Elementary Analysis of Organic Compounds, Faculty of Science, Kyushu University. MALDI TOF MS spectra were obtained using JMS-S3000 plus (JEOL) in the spiral positive ion mode. *Trans*-2-[3-(4-*tert*-Butylphenyl)-2-methyl-2-propenylidene] malononitrile (DCTB) was used as the matrix. The ESR spectra were measured using JEOL JES-FA200. The radical purities were determined by quantitative ESR measurements using 4-hydroxy-2,2,6,6-tetramethylpiperidine 1-oxyl free radical (TEMPOL) as a standard. The ESR measurements were performed three times for each sample (n = 3). The UV-vis absorption spectra were recorded using a Shimadzu UV-2600 spectrometer with a quartz cell having a 1cm optical length at 25ºC. The PL spectra were measured using a HORIBA JOBIN YVON FluoroMax-Plus Model: KUA11 with a 475 nm-long path filter at room temperature. The photoluminescence quantum yields (PLQY) were measured using a Hamamatsu Photonics Quantaurus-QY MODEL C13534-01 absolute PL quantum yield measurement system at room temperature. The excitation wavelength was 416 nm in both PL spectra and PLQY measurements for all samples. Preparative-scale gel permeation chromatography, LC-5060 (Japan Analytical Industry Co., Ltd.), with chloroform as the eluent, and an automated flash column chromatography, Biotage Isolera Spektra, were used to isolate each compound. The PL lifetime measurements were performed using a Hamamatsu Photonics Quantaurus-Tau MODEL C16361-01 (excitation wavelength of 405 nm). All calculations were performed using the Gaussian16 program package Revision C.01.[1] For fluorescence imaging, HeLa cells were seeded in 6-well plates at a density of 1×10^4 cells per well in Dulbecco's Modified Eagle Medium (DMEM) and allowed to adhere. TTM-*tris*TP3B or TTM-*tris*TP9B was added to a final concentration of 10 μM, and the cells were incubated for 1, 3, 6, and 24 h. After incubation, the cells were washed three times with phosphate-buffered saline (PBS) and maintained in PBS for imaging. Fluorescence images were acquired using a cyanine 5 (Cy5) filter ($\lambda_{ex}/\lambda_{em}$ = 620/700 nm). Cell viability was evaluated using an MTT assay. HeLa Cells were seeded in 96-well plates at a density of 3,000 cells per well and incubated overnight. The cells were then treated with TTM-*tris*TP3B at concentrations of 0, 1, 3, 10, 30, and 100 μM for 48 h. After

treatment, MTT solution was added to each well and incubated for 4 h. The resulting formazan crystals were dissolved in DMSO, and the absorbance was measured at 570 nm. Cell viability was expressed as a percentage relative to the untreated control (0 μM), which was defined as 100%. The IC_{50} value was determined by nonlinear regression using a four-parameter logistic model. MRI data were acquired using a horizontal 1.0 T Bruker ICON MRI system (Bruker Biospin, Bruker Corporation, Ettlingen, Germany) with a dedicated solenoid coil for the phantom. For quantitative T_1 mapping, an inversion recovery (IR) sequence with RARE readout was used with the following parameters: repetition time (TR) = 12,000 ms, effective echo time (TE) = 10 ms, inversion times (TI) = 20, 28, 39, 55, 77, 108, 151, 211, 295, 413, 579, 810, 1134, 1587, 2222, 3111, 4356, 6098, 8538, and 11,953 ms, RARE factor = 4, number of averages = 1, slice thickness = 3 mm, field of view (FOV) = 48 × 19.2 mm^2, and matrix size = 125 × 50. T_1-weighted images were acquired using the RARE sequence with the following parameters: repetition time (TR) = 600 ms, effective echo time (TE) = 16 ms, RARE factor = 2, number of averages = 4, slice thickness = 3 mm, field of view (FOV) = 48 × 19.2 mm^2, and matrix size = 200 × 80. Dynamic light scattering (DLS) experiments were performed using an ELSZneo (Otsuka Electronics Co., Ltd., Japan) at 25 °C under a scattering angle of 165° at λ = 638 nm. To avoid the influence of radical emission on the experimental results, the measurements were performed using the closed-shell radical precursors. The blank sample was prepared by filtering Milli-Q water through a 0.45 μm pore-size filter. Radical precursor aqueous solutions were prepared to be 10^{-5}, 10^{-4}, and 10^{-3} M using the same batch of Milli-Q water as the blank sample, and filtered through a 0.45 μm pore-size filter before the measurements. The particle size distributions were analyzed by the Marquardt method. Molecular structures were drawn using ChemDraw22.2.0, graphs were prepared using Microsoft Excel, and schematic illustrations and graphical abstract were created using Microsoft PowerPoint.

Synthesis

1-(4,4,5,5-Tetramethyl-1,3,2-dioxaborolan-2-yl)-2,4,6-trimethoxybenzene (TMB-Bpin)

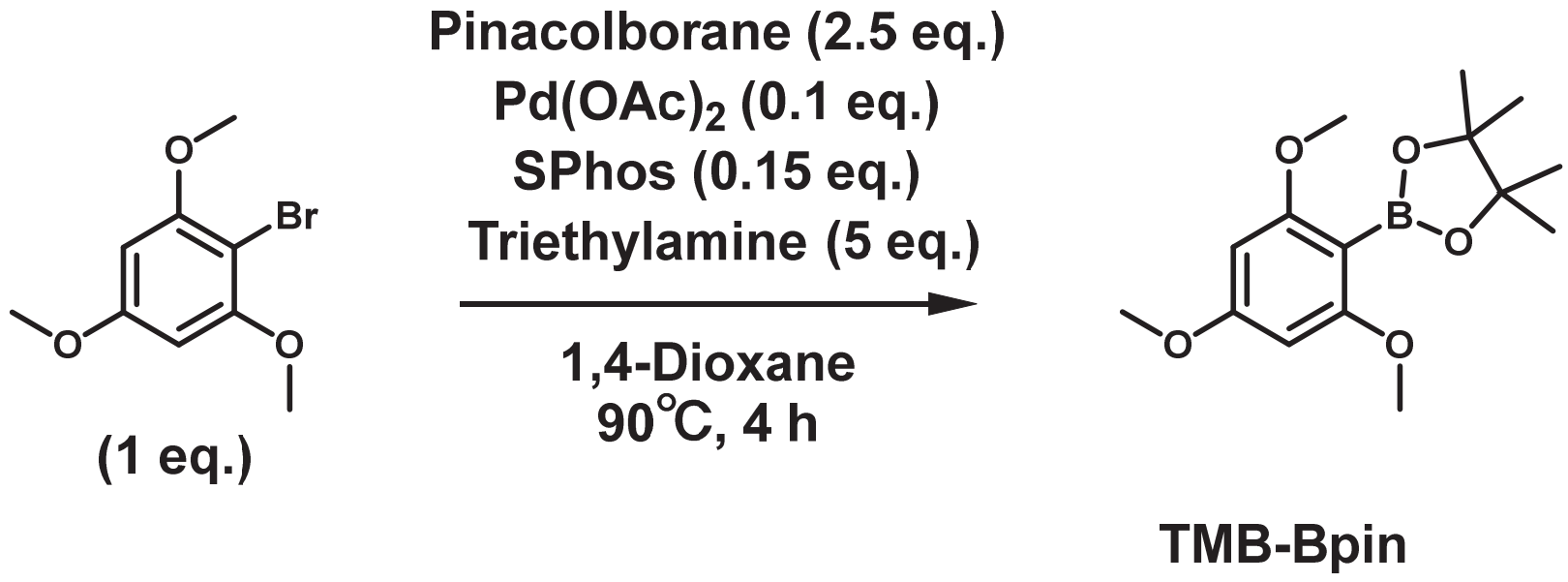

Under nitrogen atmosphere, a mixture of 1-bromo-2,4,6-trimethoxybenzene (25.01 g, 0.10 mol), pinacolborane (36.4 mL, 0.25 mol), $Pd(OAc)_2$ (2.29 g, 10.16 mmol), SPhos (6.25 g, 15.22 mmol), triethylamine (70.1 mL, 0.51 mol), and anhydrous 1,4-dioxane (250 mL) was added into a 3-neck flask and stirred at 90°C for 4 h. After the solution cooled to room temperature, the mixture was filtered through Celite. The residue was extracted with ethyl acetate and washed with brine. The combined organic solution was dried over Na_2SO_4 and concentrated. The crude was purified by silica gel column chromatography (eluent: chloroform) and recrystallized (ethyl acetate/hexane) to obtain TMB-Bpin as a colorless powder (20.59g, 69%).

The ^{1}H NMR spectrum was identical to the previous report.[2]

^{1}H-NMR (400 MHz, $CDCl_3$) δ 6.04 (s, 2H), 3.79 (s, 3H), 3.74 (s, 6H), 1.36 (s, 12H)

HTTM-*tris*TMB

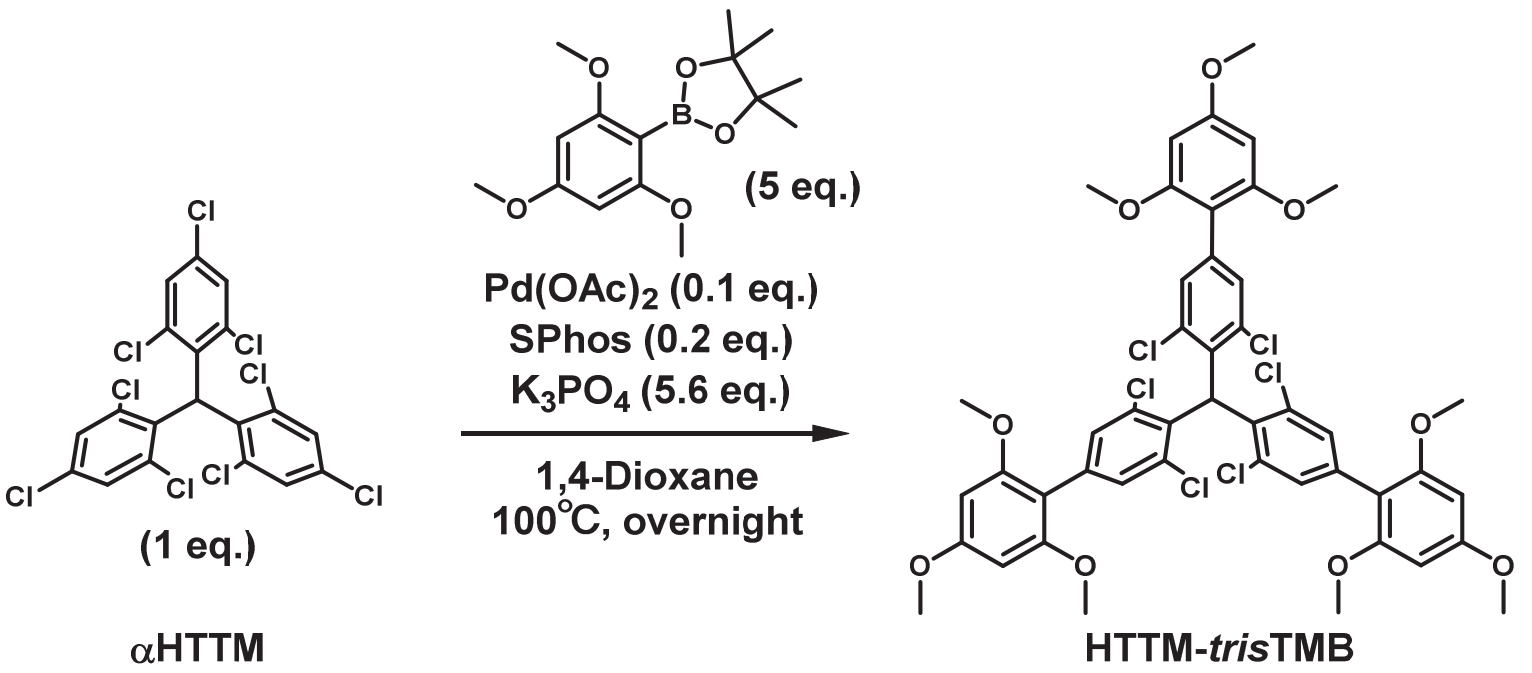

Under nitrogen atmosphere, a mixture of 1-(4,4,5,5-Tetramethyl-1,3,2-dioxaborolan-2-yl)-2,4,6-trimethoxy benzene (7.97g, 27.10 mmol), αHTTM (3.00g, 5.41 mmol), $Pd(OAc)_2$ (0.25g, 1.11 mmol), SPhos (0.70g, 1.71 mmol), K_3PO_4 (11.68g, 55.02 mmol), anhydrous 1,4-dioxane (54 mL) was added into a 3-neck flask and stirred at 100°C for overnight. After the solution cooled to room temperature, the mixture was filtered through Celite. The residue was extracted with ethyl acetate and washed with pure water. The organic layer was concentrated, and the crude was roughly purified by silica gel column chromatography (eluent: chloroform). The roughly isolated crude was reprecipitated (chloroform/hexane) to obtain HTTM-*tris*TMB as a colorless powder (0.42 g, 8%).

^{1}H-NMR (400 MHz, $CDCl_3$) δ 7.32 (d, J = 1.4 Hz, 3H), 7.19 (d, J = 1.4 Hz, 3H), 7.01 (s, 1H), 6.19 (s, 6H), 3.85 (s, 9H), 3.73 (s, 18H)

^{13}C-NMR (151 MHz, $CDCl_3$) δ 161.0, 158.4, 136.7, 135.8, 134.8, 133.9, 132.8, 130.9, 109.9, 91.0, 56.0, 55.4, 50.5

MALDI TOF MS

$[M]^+$ (m/z) Calcd for: 948.0775; Found 948.4006

Elemental analysis

Calcd for $C_{46}H_{40}Cl_6O_9$, C: 58.19, H: 4.25; Found, C: 58.39, H: 4.44

HTTM-*tris*THB

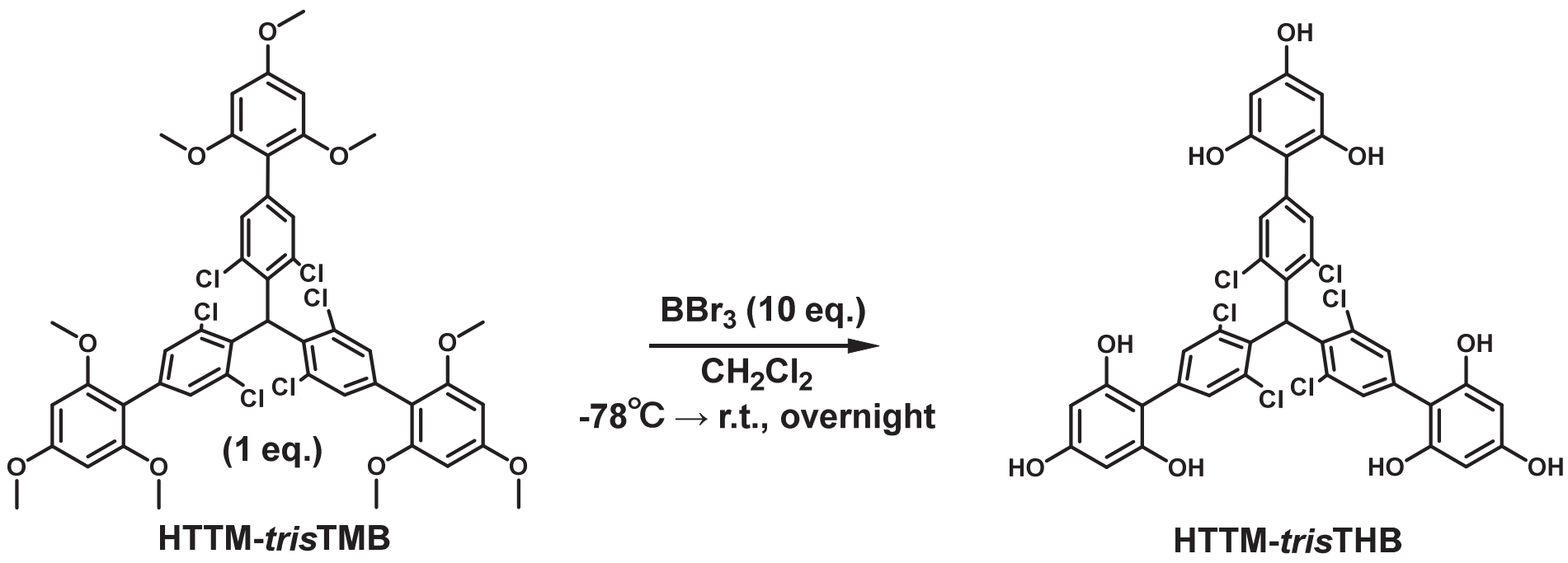

Under a nitrogen atmosphere and in the dark, a mixture of HTTM-*tris*TMB (0.35 g, 0.37 mmol) and anhydrous CH_2Cl_2 (7 mL) was added into a 2-neck flask, and then BBr_3 (1 M CH_2Cl_2 solution) (3.7 mL) was added slowly at -78°C and stirred at room temperature overnight. The mixture was slowly poured into ice-water to quench BBr_3. The residue was extracted with ethyl acetate and washed with pure water. The organic layer was concentrated, and the crude was purified by silica gel column chromatography (eluent: chloroform/methanol) to obtain HTTM-*tris*THB as a pale brown powder (0.18 g, 60%).

^{1}H-NMR (600 MHz, DMSO-D6) δ 9.34 (s, 6H), 9.30 (s, 3H), 7.42 (d, J = 1.8 Hz, 3H), 7.27 (d, J = 1.8 Hz, 3H), 6.79 (s, 1H), 5.92 (s, 6H)

^{13}C-NMR (151 MHz, DMSO-D6) δ 158.3, 156.2, 136.6, 135.1, 134.3, 132.5, 131.8, 130.8, 104.1, 94.7, 49.8, 39.5

MALDI TOF MS

$[M]^+$ (m/z) Calcd for: 821.9365; Found 821.7735

Elemental analysis

Calcd for $C_{37}H_{22}Cl_6O_9$ [$+2H_2O$], C: 51.72, H: 3.05; Found, C: 51.84, H: 3.29

2-(2-(2-methoxyethoxy)ethoxy)ethyl 4-methylbenzenesulfonate (Ts-mPEG3)

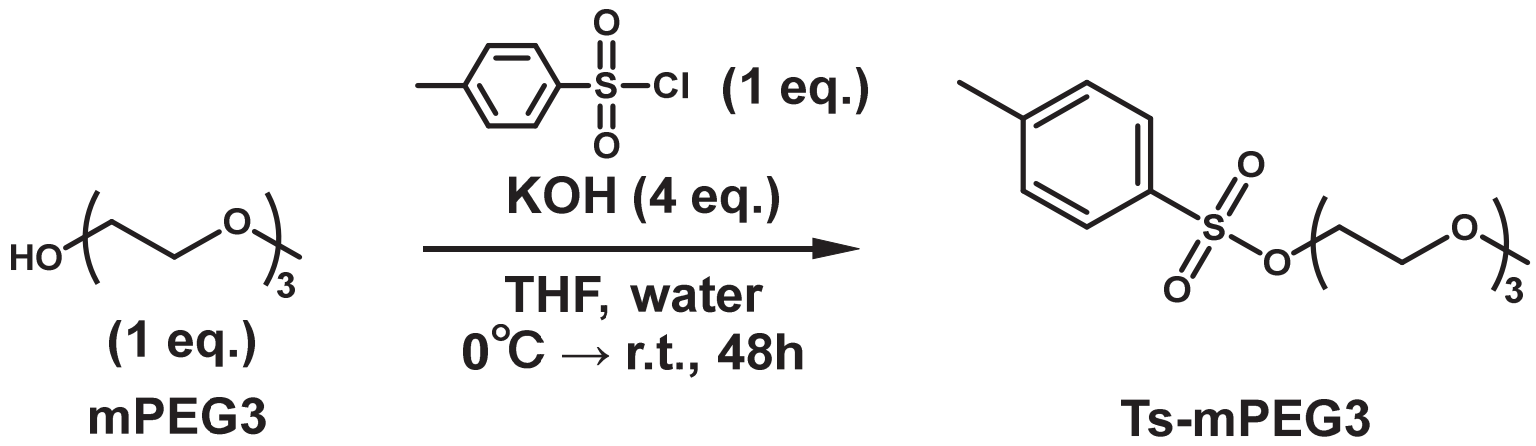

A mixture of triethylene glycol monomethyl ether (mPEG3) (5.00 g, 30.45 mmol) and THF (38 mL) was added to a round-bottom flask and stirred at 0°C for 10 min, and then KOH (8 M aqueous solution) (15.2 mL) was added slowly and stirred at 0°C for 10 min. Subsequently, *p*-toluenesulfonyl chloride (5.84 g, 30.63 mmol) was added and stirred at room temperature for 2 days. The mixture was extracted with ethyl acetate and washed with pure water. The combined organic solution was dried over Na_2SO_4 and concentrated to give the crude product as a colorless viscous liquid. The crude was purified by silica gel column chromatography (chloroform: methanol = 19: 1) to obtain Ts-mPEG3 as a colorless viscous liquid (8.29 g, 86 %).

The ^{1}H NMR spectrum was identical to the previous report.[3]

^{1}H-NMR (400 MHz, $CDCl_3$) δ 7.80 (d, J = 8.2 Hz, 2H), 7.34 (d, J = 8.2 Hz, 2H), 4.16 (t, J = 5.0 Hz, 2H), 3.69 (t, J = 4.8 Hz, 2H), 3.62-3.52 (m, 8H), 3.37 (s, 3H), 2.45 (s, 3H)

2,5,8,11,14,17,20,23,26-nonaoxaoctacosan-28-yl 4-methylbenzenesulfonate (Ts-mPEG9)

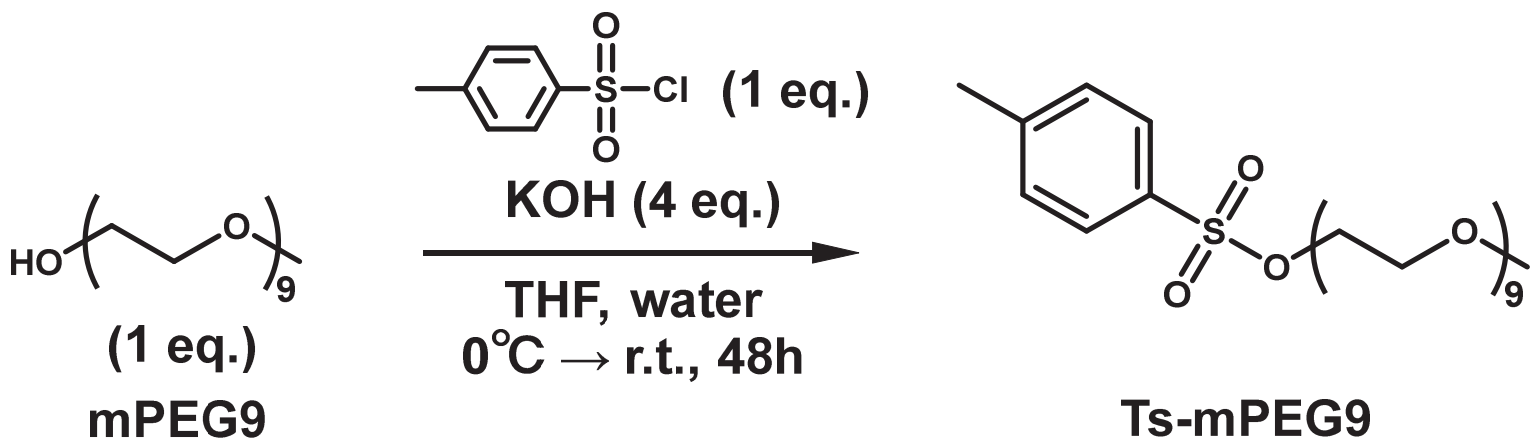

A mixture of nonaethylene glycol monomethyl ether (mPEG9) (5.00 g, 11.67 mmol) and THF (20 mL) was added to a round-bottom flask and stirred at 0°C for 10 min, and then KOH (8 M aqueous solution) (5.8 mL) was added slowly and stirred at 0°C for 10 min. Subsequently, *p*-toluenesulfonyl chloride (2.30 g, 12.06 mmol) was added and stirred at room temperature for 2 days. The mixture was extracted with ethyl acetate and washed with pure water. The combined organic solution was dried over Na_2SO_4 and concentrated to give the crude product as a colorless viscous liquid. The crude was purified by silica gel column chromatography (chloroform: methanol = 19: 1) to obtain Ts-mPEG9 as a colorless viscous liquid (6.18 g, 91 %).

The ^{1}H NMR spectrum was identical to the previous report.[4]

^{1}H-NMR (400 MHz, $CDCl_3$) δ 7.80 (dt, J = 8.7, 1.8 Hz, 2H), 7.35 (d, J = 8.2 Hz, 2H), 4.17-3.38 (m, 39H), 2.45 (s, 3H)

HTTM-*tris*TP3B

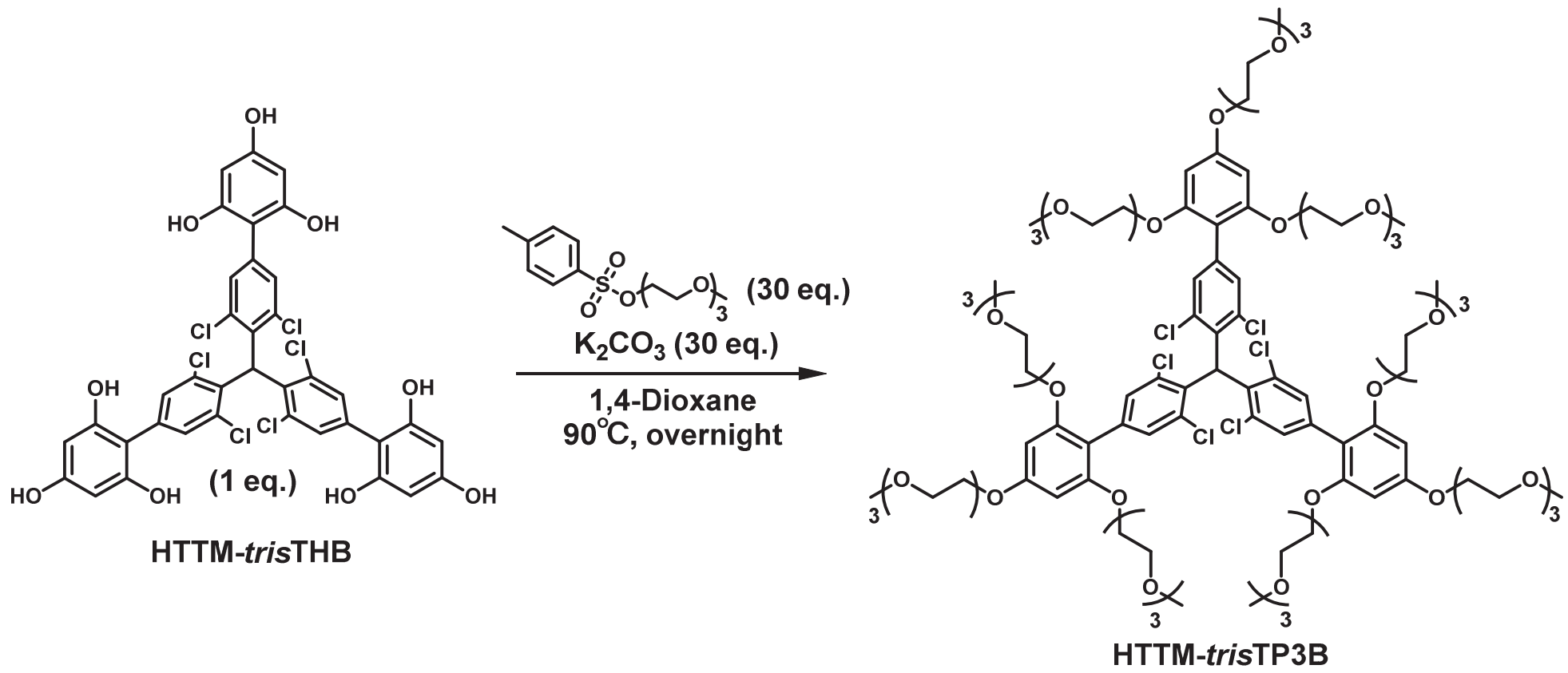

Under a nitrogen atmosphere, a mixture of HTTM-*tris*THB (0.08 g, 0.1 mmol), Ts-mPEG3 (0.93 g, 2.92 mmol), K_2CO_3 (0.41 g, 2.97 mmol), and anhydrous 1,4-dioxane (4 mL) was added to a 2-neck flask and stirred at 90°C overnight. After the solution cooled to room temperature, the mixture was transferred to the eggplant flask and concentrated. The crude was dissolved in chloroform and filtered to remove the base. The residue was concentrated and purified by preparative column gel permeation chromatography (eluent: chloroform) to obtain HTTM- *tris*TP3B as a pale brown viscous liquid (0.13 g, 63%).

^{1}H-NMR (600 MHz, $CDCl_3$) δ 7.38 (d, J = 1.7 Hz, 3H), 7.23 (d, J = 1.7 Hz, 3H), 6.89 (s, 1H), 6.20 (s, 6H), 4.14-4.13 (m, 6H), 4.06-3.98 (m, 12H), 3.88-3.86 (m, 6H), 3.76-3.74 (m, 6H), 3.73-3.48 (m, 78H), 3.36 (d, J = 37.1 Hz, 27H)

^{13}C-NMR (151 MHz, $CDCl_3$) δ 160.2, 157.4, 136.2, 135.3, 134.9, 133.6, 132.9, 131.1, 110.5, 93.2, 71.9, 71.8, 71.1, 70.9, 70.8, 70.8, 70.7, 70.7, 70.6, 70.5, 70.5, 70.4, 69.8, 69.7, 69.6, 68.8, 67.5, 59.1, 59.0, 59.0, 50.5

MALDI TOF MS

$[M+Na]^+$ (m/z) Calcd for: 2161.7764; Found: 2161.9072

Elemental analysis

Calcd for $C_{100}H_{148}Cl_6O_{36}$ [+2H_2O], C: 55.22, H: 7.04; Found, C: 55.16, H: 6.95

HTTM-*tris*TP9B

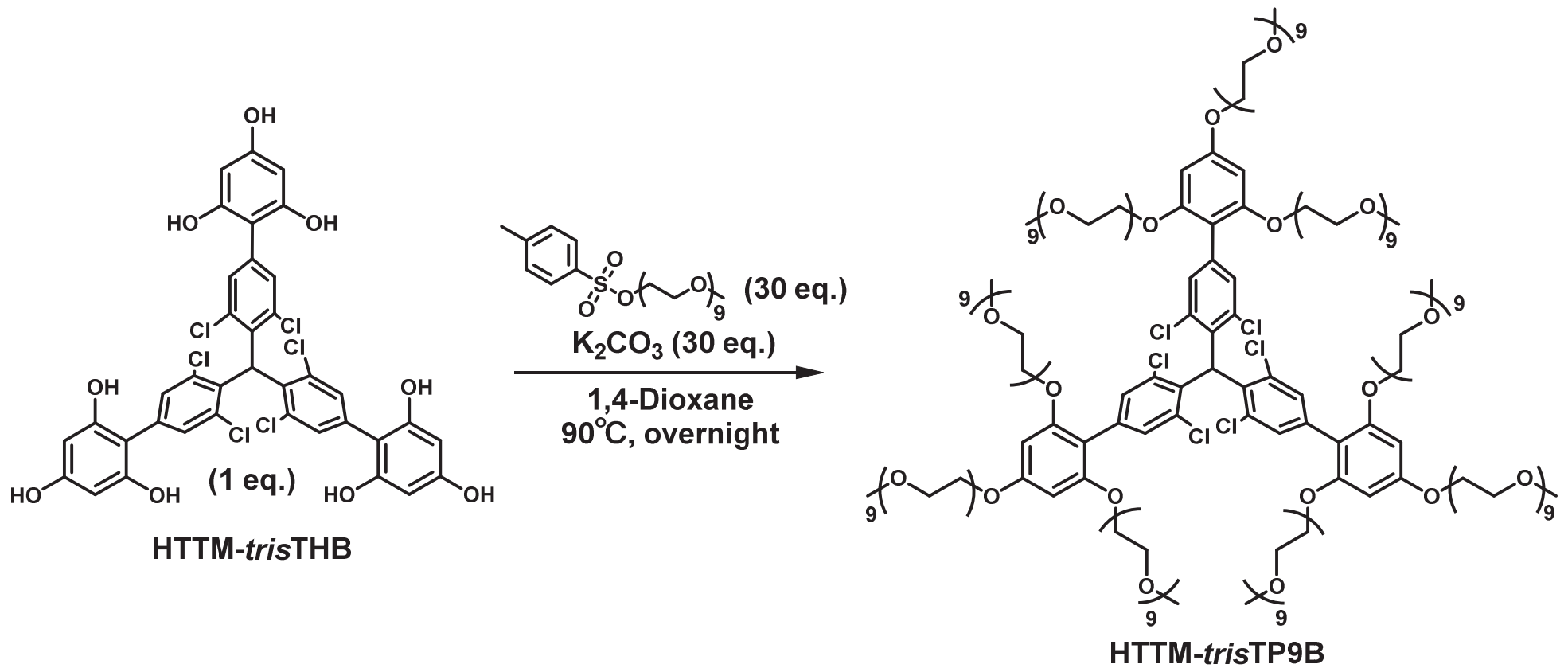

Under a nitrogen atmosphere, a mixture of HTTM-*tris*THB (0.08 g, 0.1 mmol), Ts-mPEG9 (1.71 g, 2.93 mmol), K_2CO_3 (0.41 g, 2.97 mmol), and anhydrous 1,4-dioxane (4 mL) was added to a 2-neck flask and stirred at 90°C overnight. After the solution cooled to room temperature, the mixture was transferred to the eggplant flask and concentrated. The crude was dissolved in chloroform and filtered to remove the base. The residue was concentrated and purified by preparative column gel permeation chromatography (eluent: chloroform) to obtain HTTM- *tris*TP9B as a pale brown viscous liquid (0.19 g, 44%).

^{1}H-NMR (600 MHz, $CDCl_3$) δ 7.36 (d, J = 1.7 Hz, 3H), 7.22 (d, J = 1.6 Hz, 3H), 6.87 (s, 1H), 6.20 (s, 6H), 4.13-4.12 (m, 6H), 4.05-3.98 (m, 12H), 3.86 (t, J = 4.8 Hz, 6H), 3.75-3.73 (m, 6H), 3.70-3.54 (m, 294H), 3.38 (d, J = 2.8 Hz, 27H)

^{13}C-NMR (151 MHz, $CDCl_3$) δ 160.2, 157.4, 136.1, 135.3, 134.9, 133.6, 132.9, 131.1, 128.3, 126.1, 110.5, 93.3, 71.9, 71.1, 70.9, 70.8, 70.7, 70.6, 70.6, 70.5, 70.5, 70.4, 70.4, 70.3, 69.6, 69.5, 68.8, 67.5, 59.0, 50.5

MALDI TOF MS

$[M+Na]^+$ (m/z) Calcd for: 4540.1972; Found 4540.6353

Elemental analysis

Calcd for $C_{208}H_{1364}Cl_6O_{90}$ [+$2H_2O$], C: 54.86, H: 8.15; Found, C: 54.66, H: 8.08

TTM-*tris*TP3B

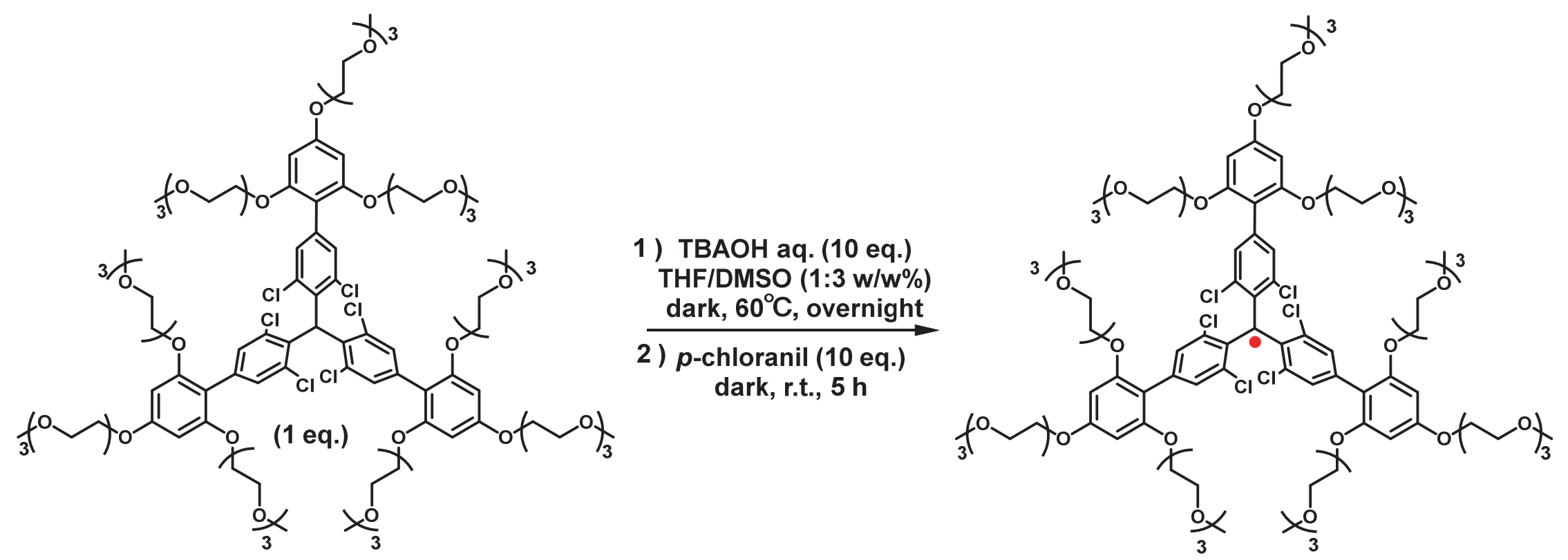

Under nitrogen atmosphere, a mixture of HTTM-*tris*TP3B (0.10 g, 0.05 mmol), anhydrous THF (4.8 mL), and anhydrous DMSO (14.3 mL) (THF: DMSO = 1:3 w/w) was added into a 3-neck flask and stirred at room temperature for 10 min. 40% tetrabutylammonium hydroxide (TBAOH) aqueous solution (0.30 mL, 0.47 mmol) was added and stirred at 60℃ for 1 day. After the solution cooled to room temperature, *p*-chloranil (0.12 g, 0.49 mmol) was added and stirred at room temperature for 5 hours. The mixture was transferred to the eggplant flask and concentrated. The crude product was dissolved in chloroform and filtered to remove the base and *p*-chloranil. The residue was concentrated and purified by preparative column gel permeation chromatography (eluent: chloroform) to obtain TTM- *tris*TP3B as a dark green viscous liquid (57 mg, 57%).

MALDI TOF MS

$[M]^+$ (m/z) Calcd for: 2137.7788; Found: 2137.9241

Elemental analysis

Calcd for $C_{100}H_{147}Cl_6O_{36}$ [+2H_2O], C: 55.25, H: 7.00; Found, C: 55.29, H: 6.88

TTM-*tris*TP9B

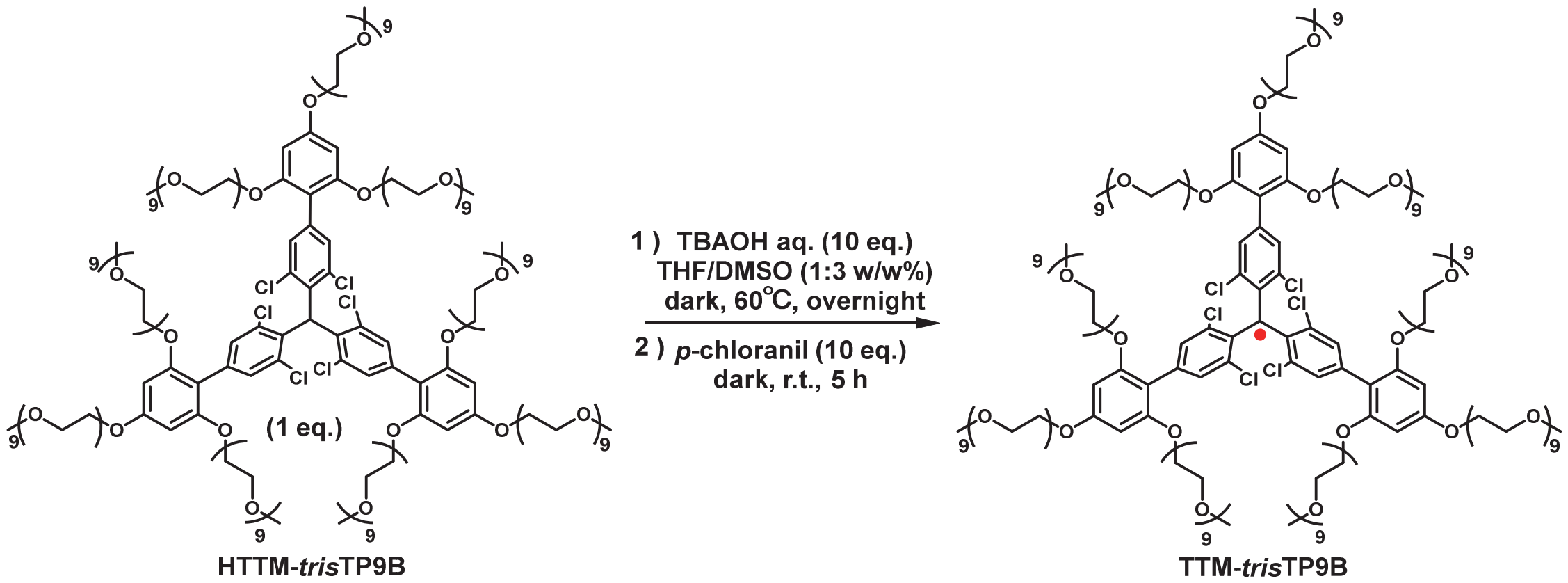

Under a nitrogen atmosphere, a mixture of HTTM-*tris*TP9B (0.10 g, 0.02 mmol), anhydrous THF (4.8 mL), and anhydrous DMSO (14.3 mL) (THF: DMSO = 1:3 w/w) was added into a 3-neck flask and stirred at room temperature for 10 min. 40% tetrabutylammonium hydroxide (TBAOH) aqueous solution (0.14 mL, 0.22 mmol) was added and stirred at 60℃ for 1 day. After the solution cooled to room temperature, *p*-chloranil (0.06 g, 0.24 mmol) was added and stirred at room temperature for 5 hours. The mixture was transferred to the eggplant flask and concentrated. The crude was dissolved in chloroform and filtered to remove the base and *p*-chloranil. The residue was concentrated and purified by preparative column gel permeation chromatography (eluent: chloroform) to obtain TTM- *tris*TP9B as a dark green viscous liquid (72 mg, 72%).

MALDI TOF MS

$[M+Na]^+$ (m/z) Calcd for: 4539.1894; Found: 4539.5039

Elemental analysis

Calcd for $C_{208}H_{363}Cl_6O_{90}$ [+2H_2O], C: 54.87, H: 8.13; Found, C: 54.88, H: 8.10

NMR spectra

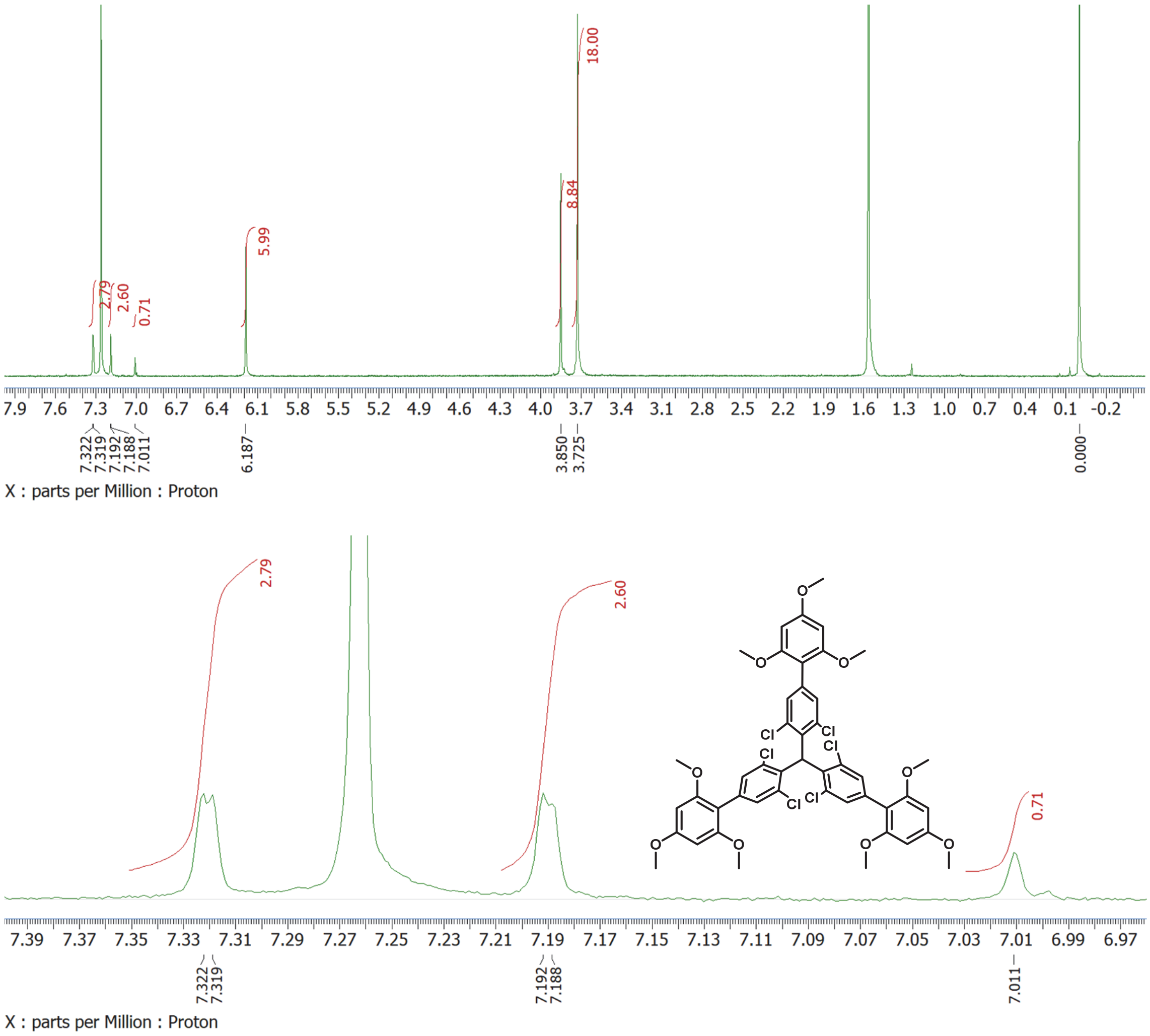

Figure S 1. ^{1}H NMR spectra of HTTM-*tris*TMB in $CDCl_3$.

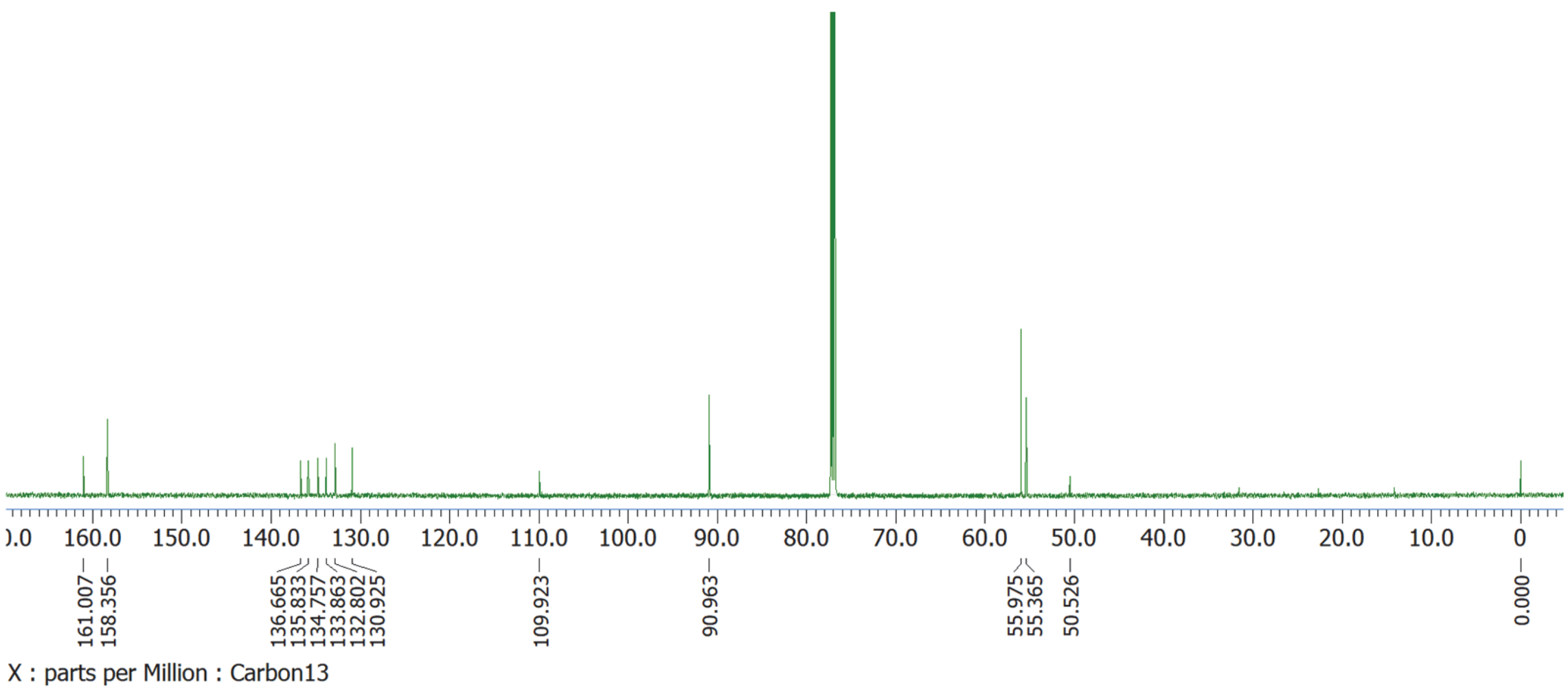

Figure S 2. ^{13}C NMR spectra of HTTM-*tris*TMB in $CDCl_3$.

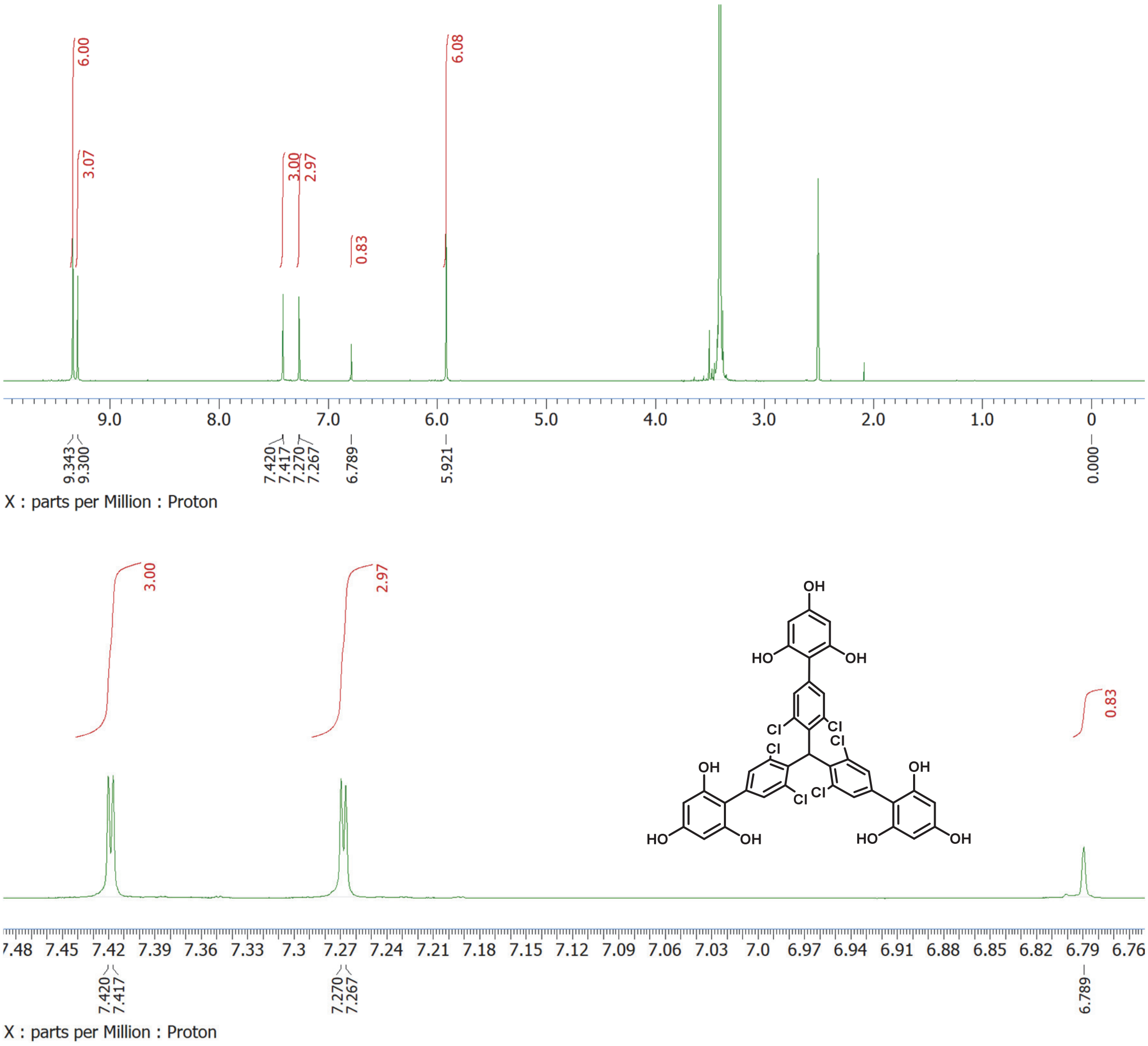

Figure S 3. ^{1}H NMR spectra of HTTM-*tris*THB in DMSO-d6.

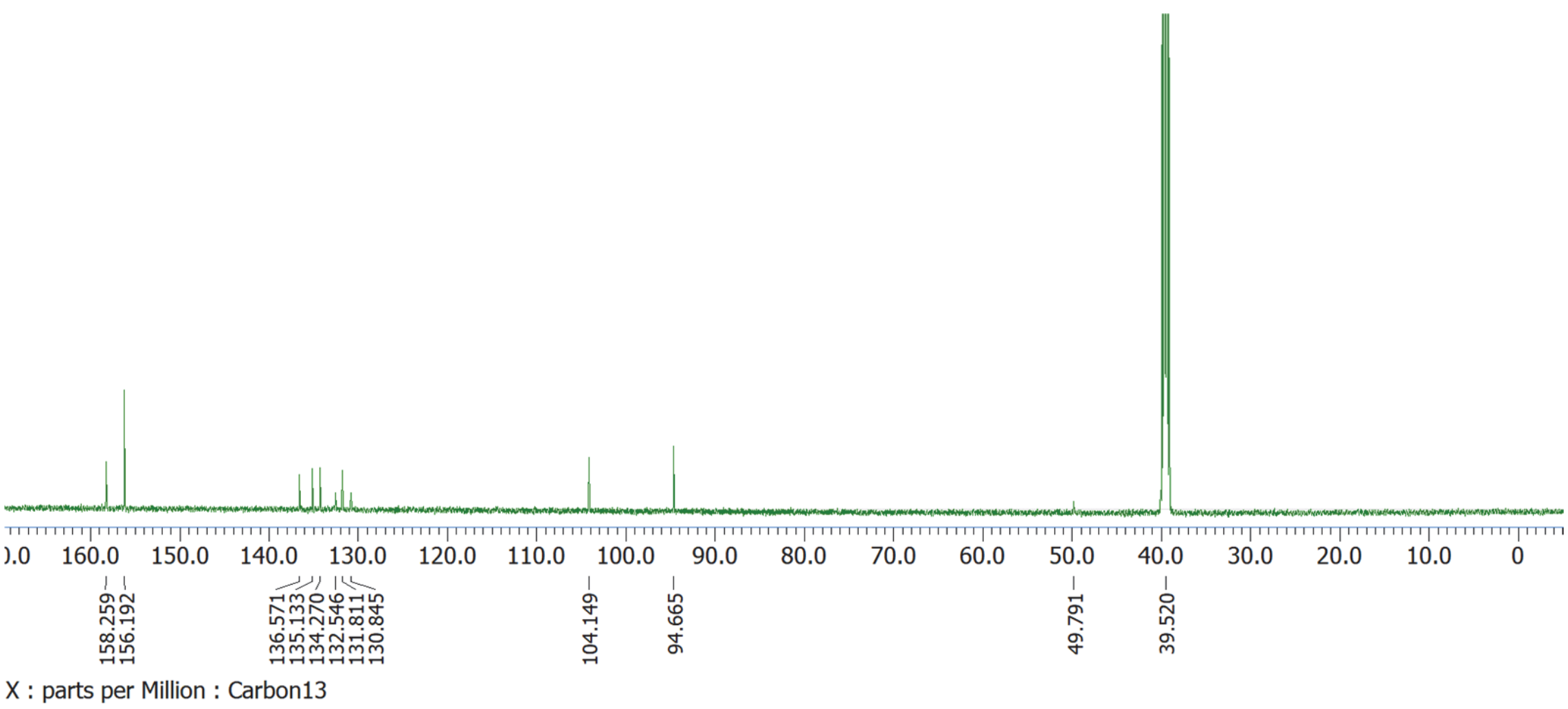

Figure S 4. ^{13}C NMR spectra of HTTM-*tris*THB in DMSO-d6.

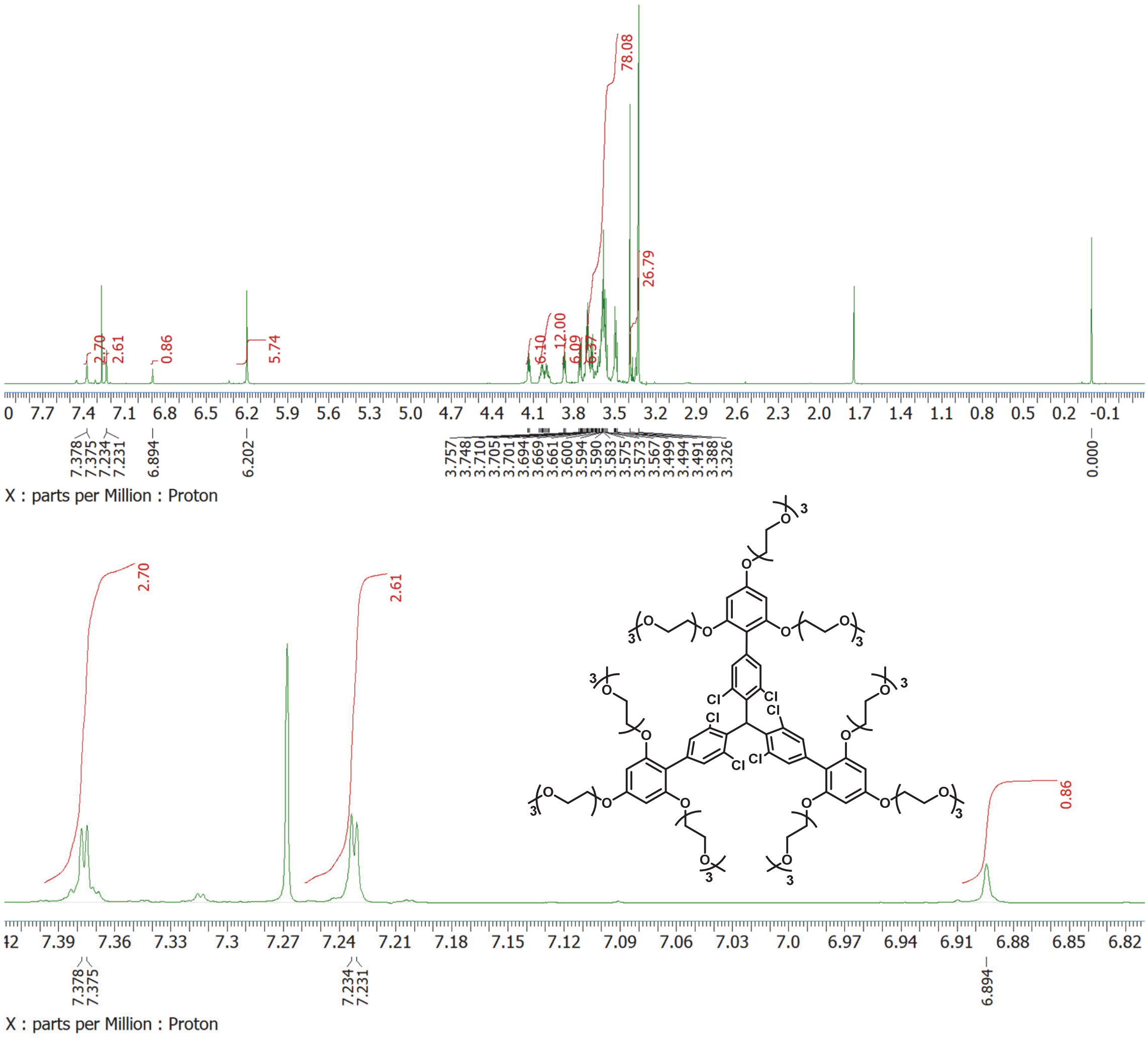

Figure S 5. ^{1}H NMR spectra of HTTM-*tris*TP3B in $CDCl_3$.

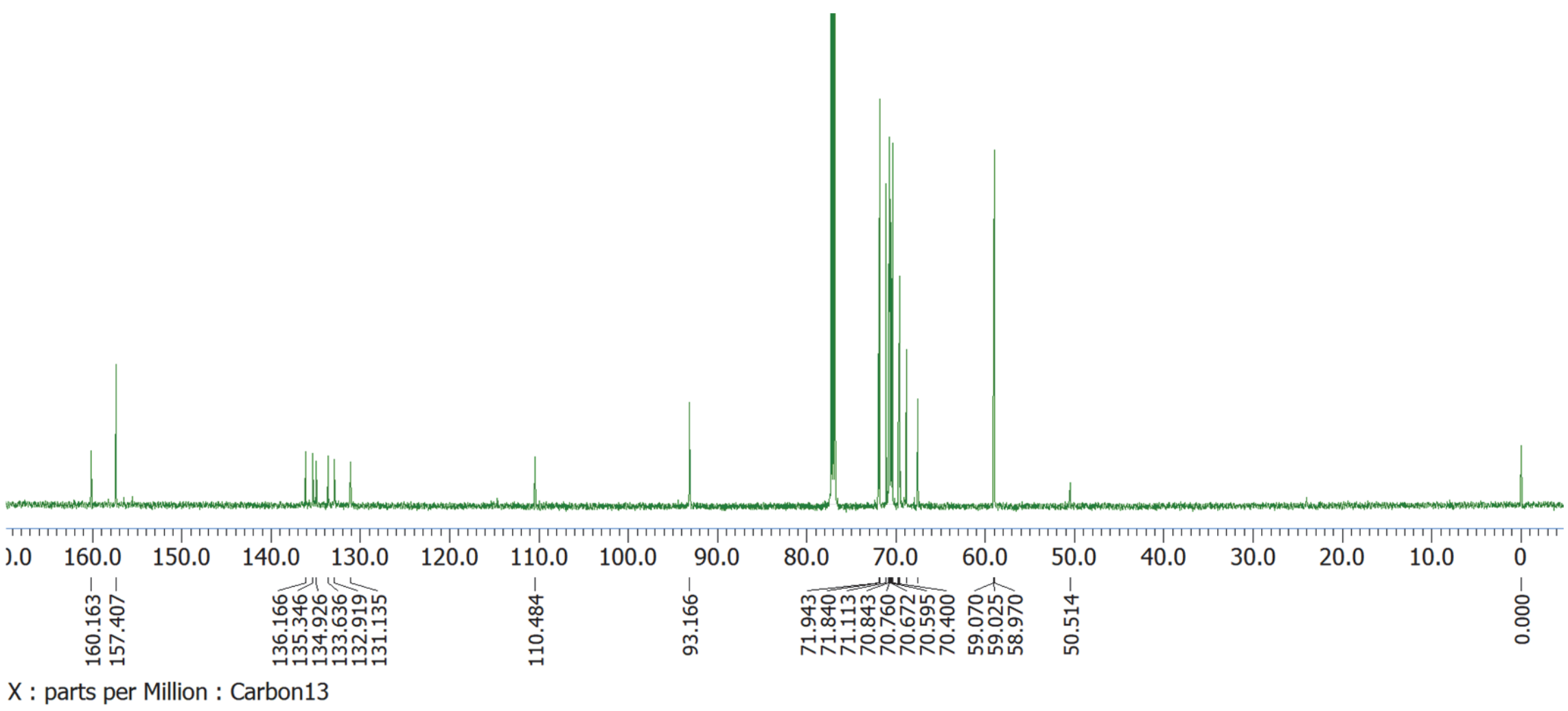

Figure S 6. ^{13}C NMR spectra of HTTM-*tris*TP3B in $CDCl_3$.

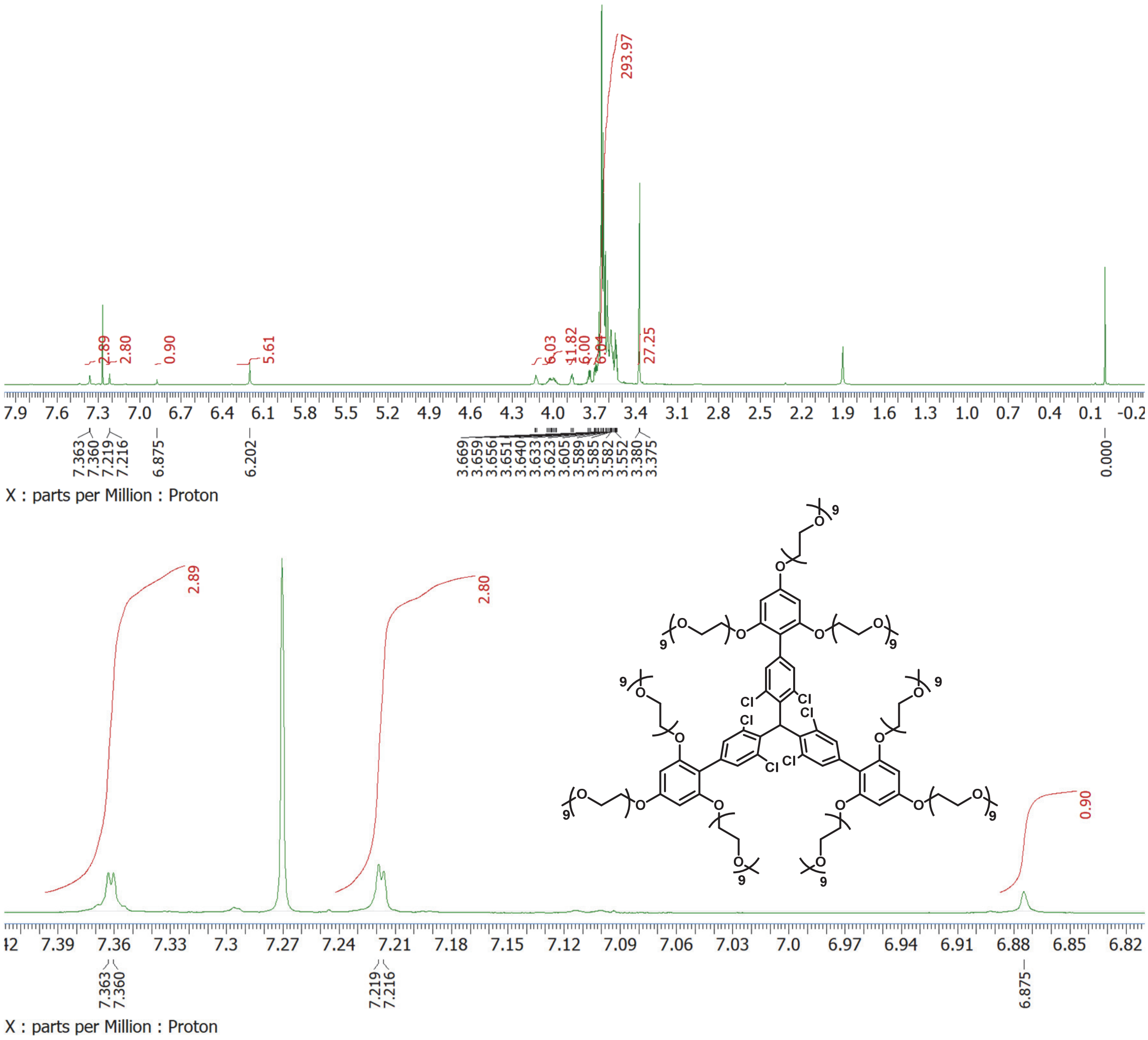

Figure S 7. ^{1}H NMR spectra of HTTM-*tris*TP9B in $CDCl_3$.

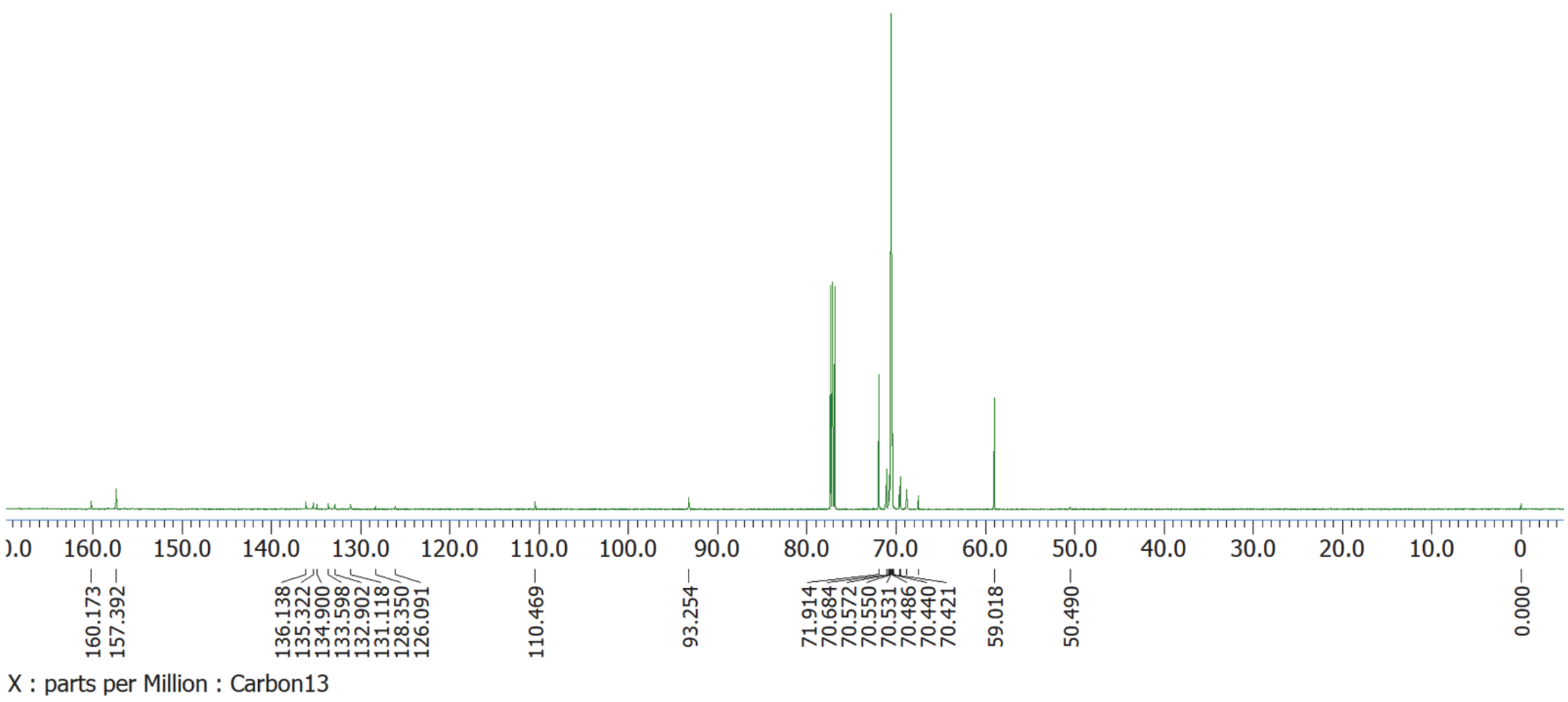

Figure S 8. ^{13}C NMR spectra of HTTM-*tris*TP9B in $CDCl_3$.

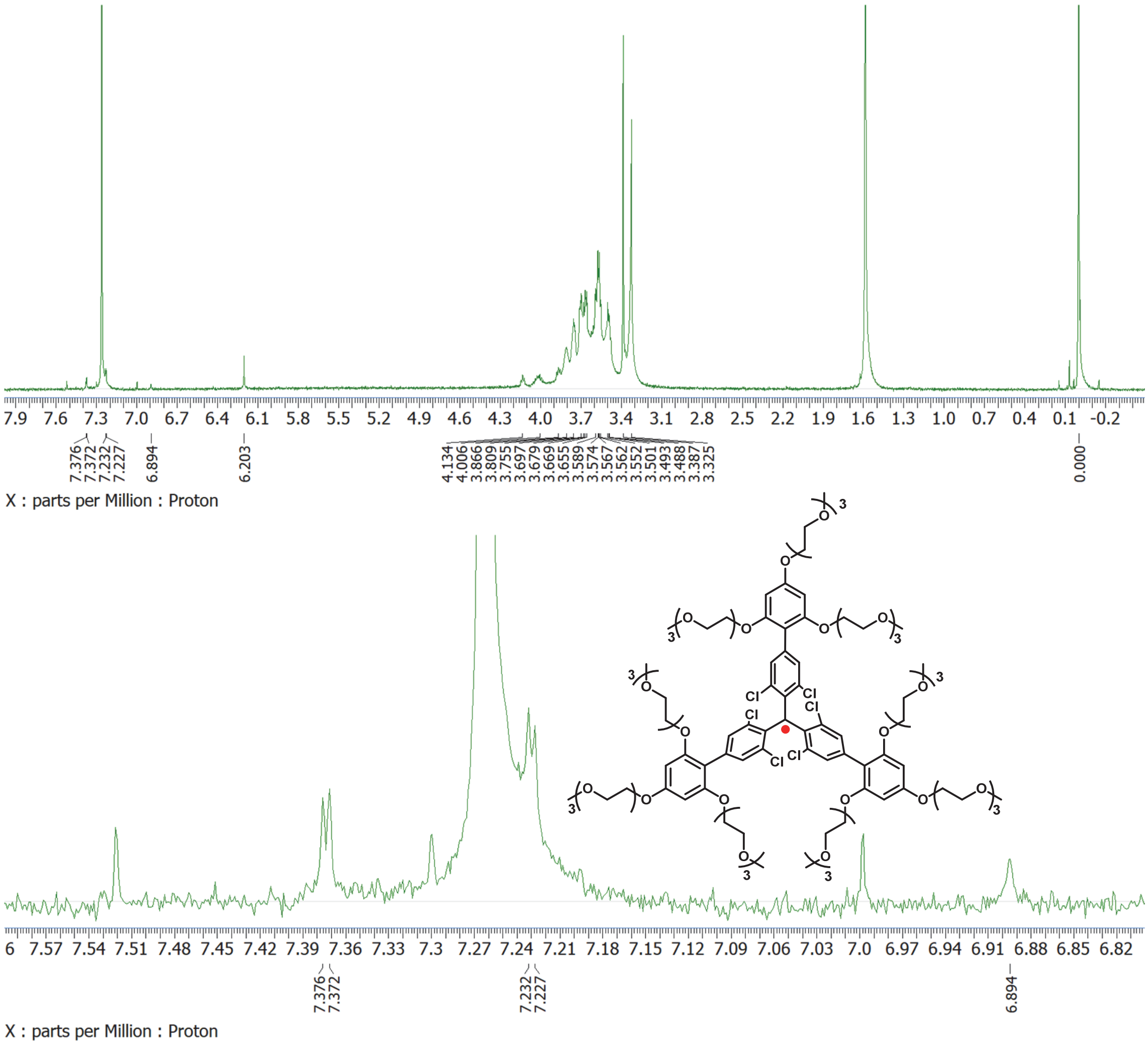

Figure S 9. ^{1}H NMR spectra of HTTM-*tris*TP3B contained in TTM-*tris*TP3B sample in $CDCl_3$.

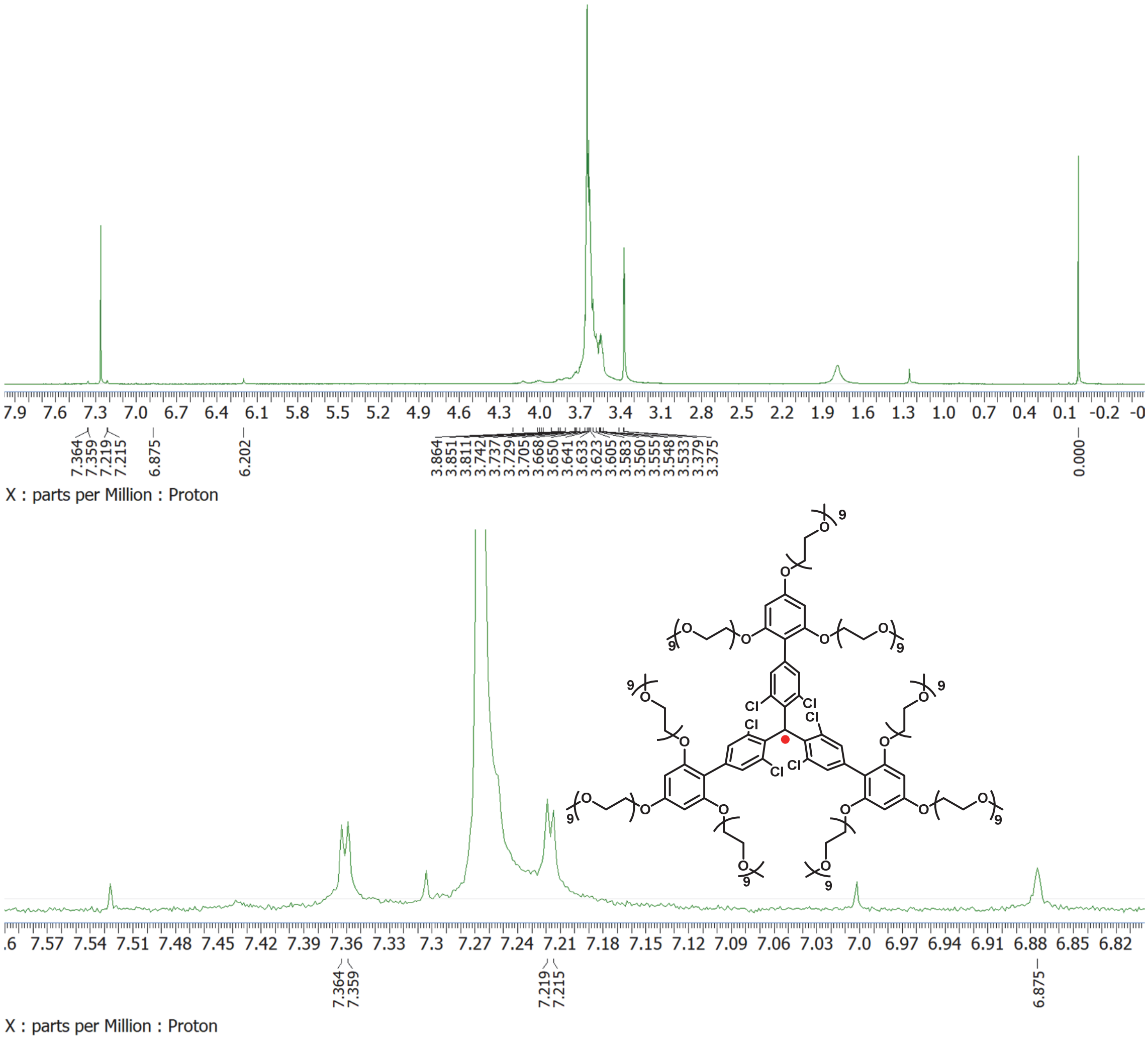

Figure S 10. ^{1}H NMR spectra of HTTM-*tris*TP9B contained in TTM-*tris*TP9B sample in $CDCl_3$.

ESR spectra

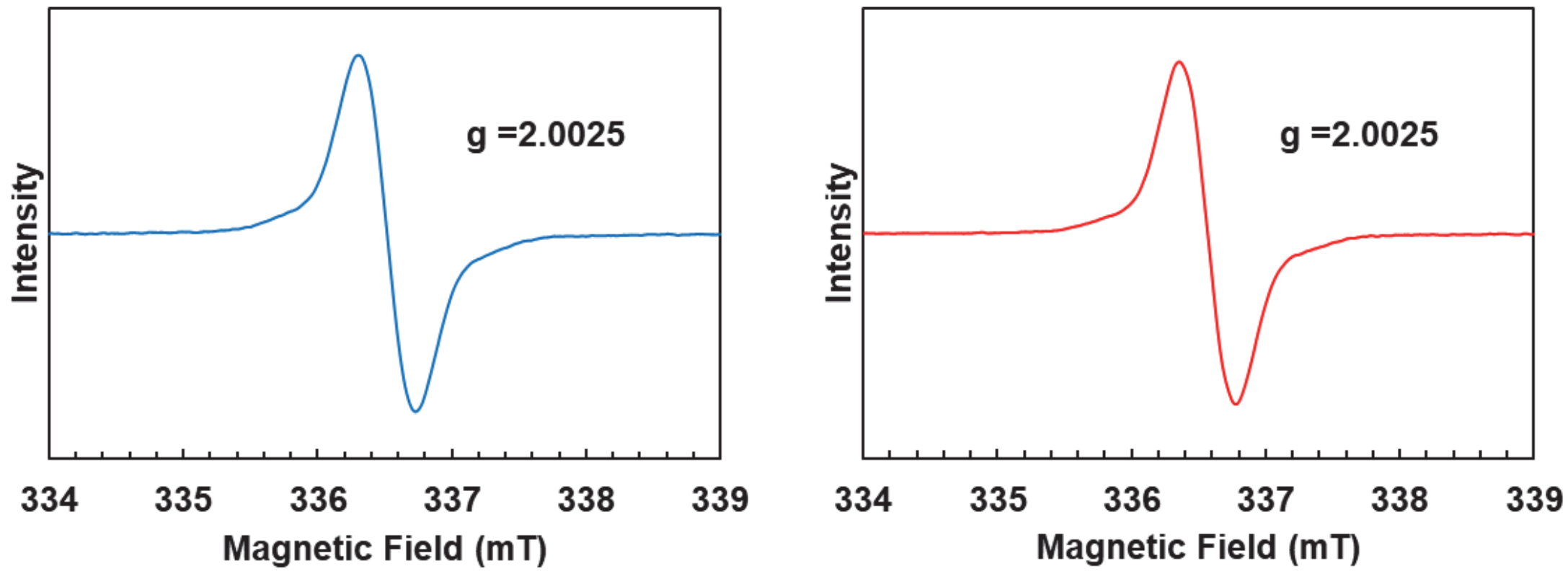

Figure S 11. ESR spectra of (left) TTM-*tris*TP3B and (red) TTM-*tris*TP9B in toluene (10^{-5} M).

The following formula was used to determine radical purity by ESR. TEMPOL was used as the standard sample. The ESR measurements for each sample were conducted three times, and the measurement errors were calculated.

$$S_x = \frac{I_x}{I_0} S_0$$

S_x: Amount of spin in TTM-*tris*TP3B and TTM-*tris*TP9B

S_0: Amount of spin in TEMPOL

I_x: Integral value of TTM-*tris*TP3B and TTM-*tris*TP9B

I_0: Integral value of TEMPOL

Table S 1. Radical purities considering the measurement errors.

data	signal int.	signal ave.	$(\text{int.–ave.})^2$	variance	SD	ave.+SD	relative error	error SD	purity (%)
TEMPOL_tol1	28.4282		0.0560						
TEMPOL_tol2	28.1453	28.1915	0.0021	0.0315	0.1775	28.1915±0.1775	3.96E-05	-	<97
TEMPOL_tol3	28.0009		0.0363						
TP3B_tol1	18.1056		0.0501						
TP3B_tol2	18.3471	18.3293	0.0003	0.0309	0.1759	18.3293±0.1759	9.21E-05	0.724	63.1±0.7
TP3B_tol3	18.5353		0.0424						
TP9B_tol1	15.1535		0.0170						
TP9B_tol2	15.3066	15.2838	0.0005	0.0097	0.0984	15.2838±0.0984	4.15E-05	0.474	52.6±0.5
TP9B_tol3	15.3913		0.0116						

*SD: Standard deviation

UV-vis absorption and PL spectra

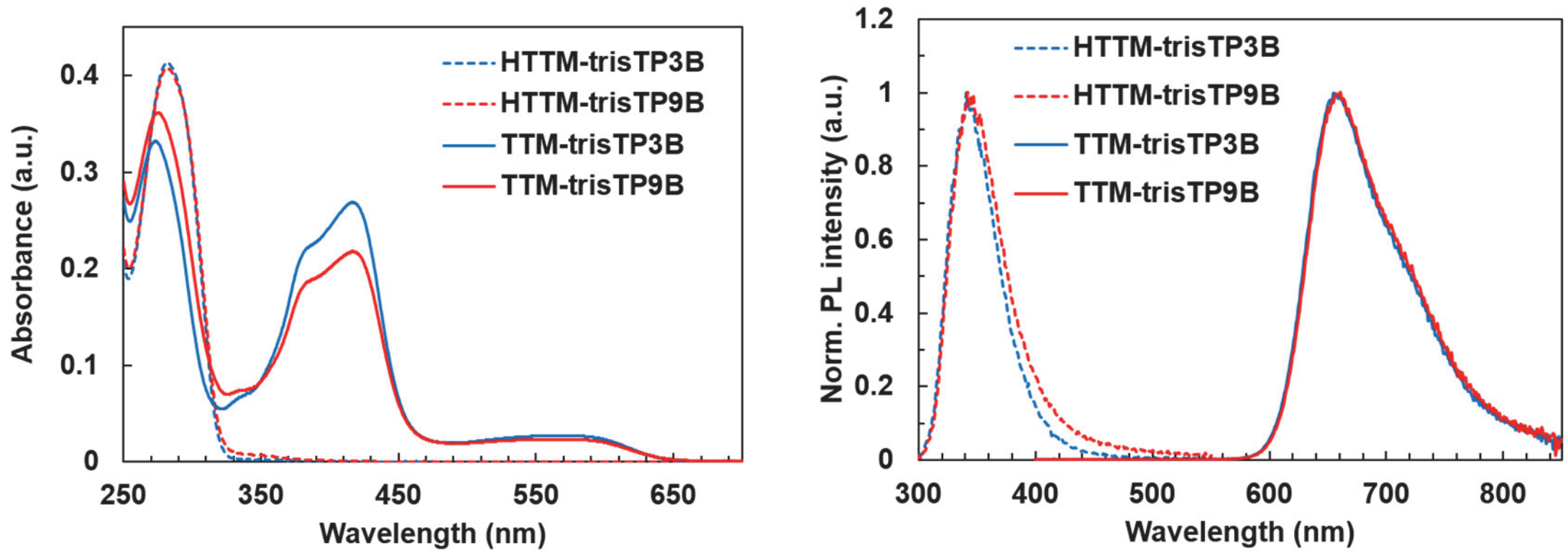

Figure S 12. (Left) UV-vis absorption and (right) PL spectra of (broken blue line) HTTM-*tris*TP3B, (broken red line) HTTM-*tris*TP9B, (solid blue line) TTM-*tris*TP3B, and (solid red line) TTM-*tris*TP9B in dichloromethane (10^{-5} M). HTTMs are radical precursors. The excitation wavelengths for HTTMs and radicals were 282 and 416 nm, respectively.

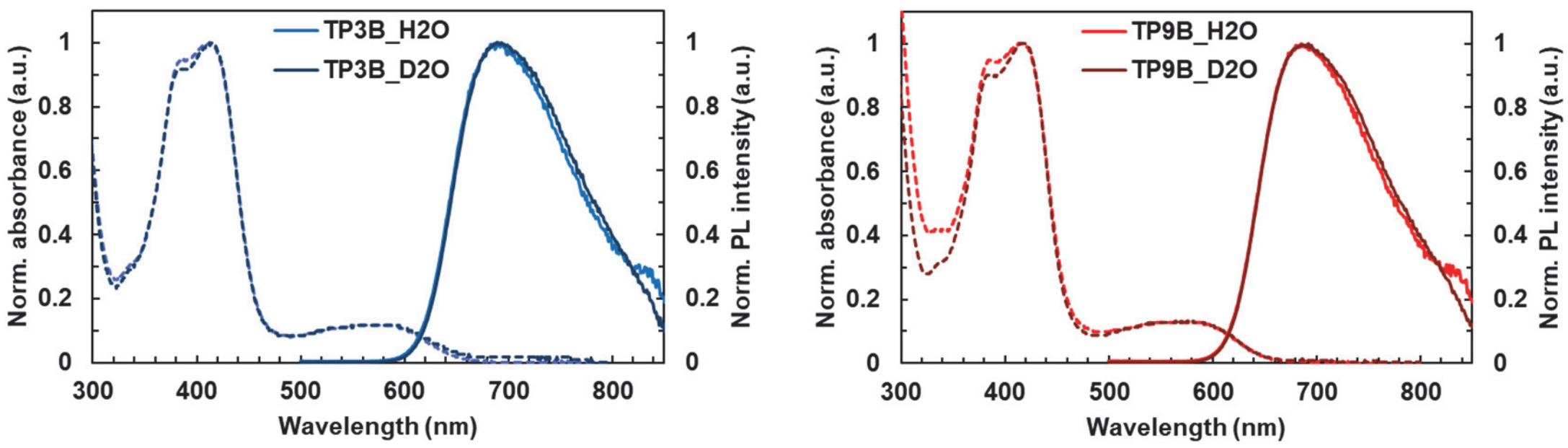

Figure S 13. UV-vis absorption and PL spectra of TTM-*tris*TP3B (TP3B) and TTM-*tris*TP9B (TP9B) in water (H2O) and deuterated water (D2O) (10^{-5} M).

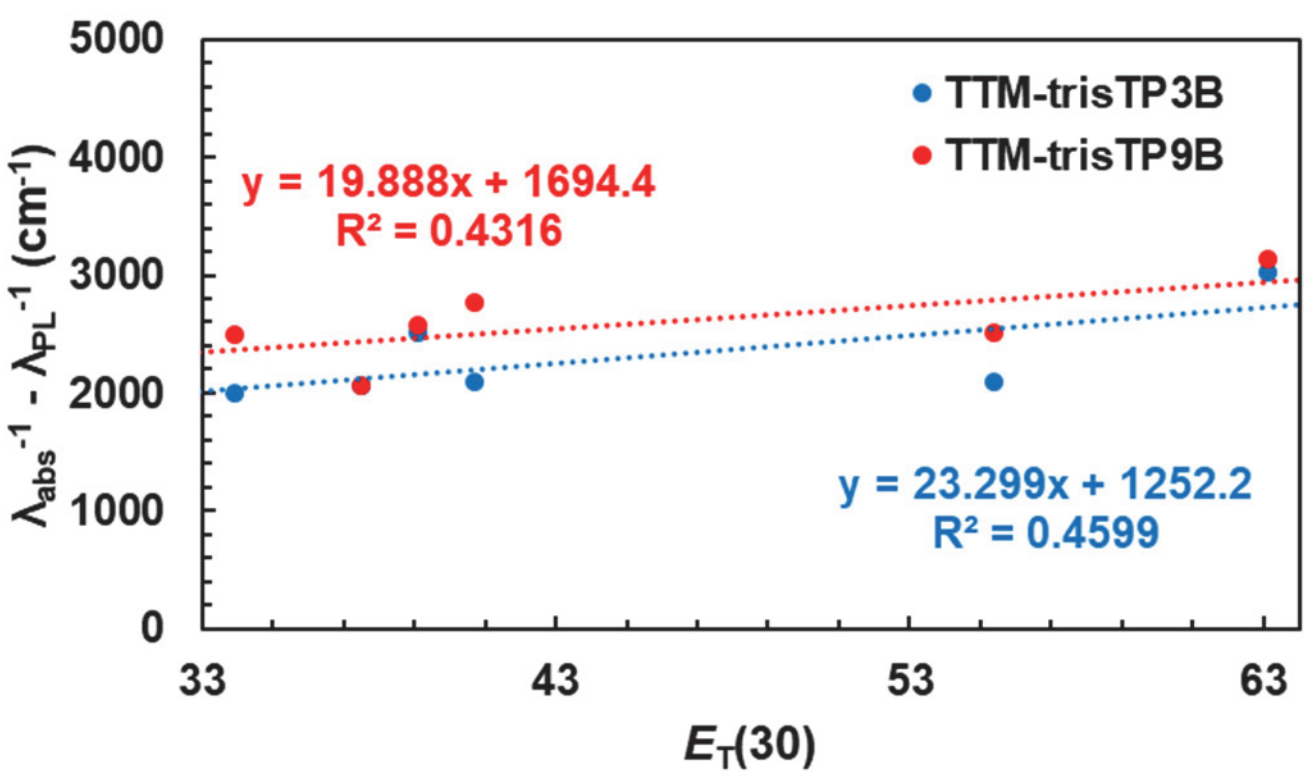

Figure S 14. Lippert-Mataga plots of TTM-*tris*TP3B and TTM-*tris*TP9B.

PL lifetime

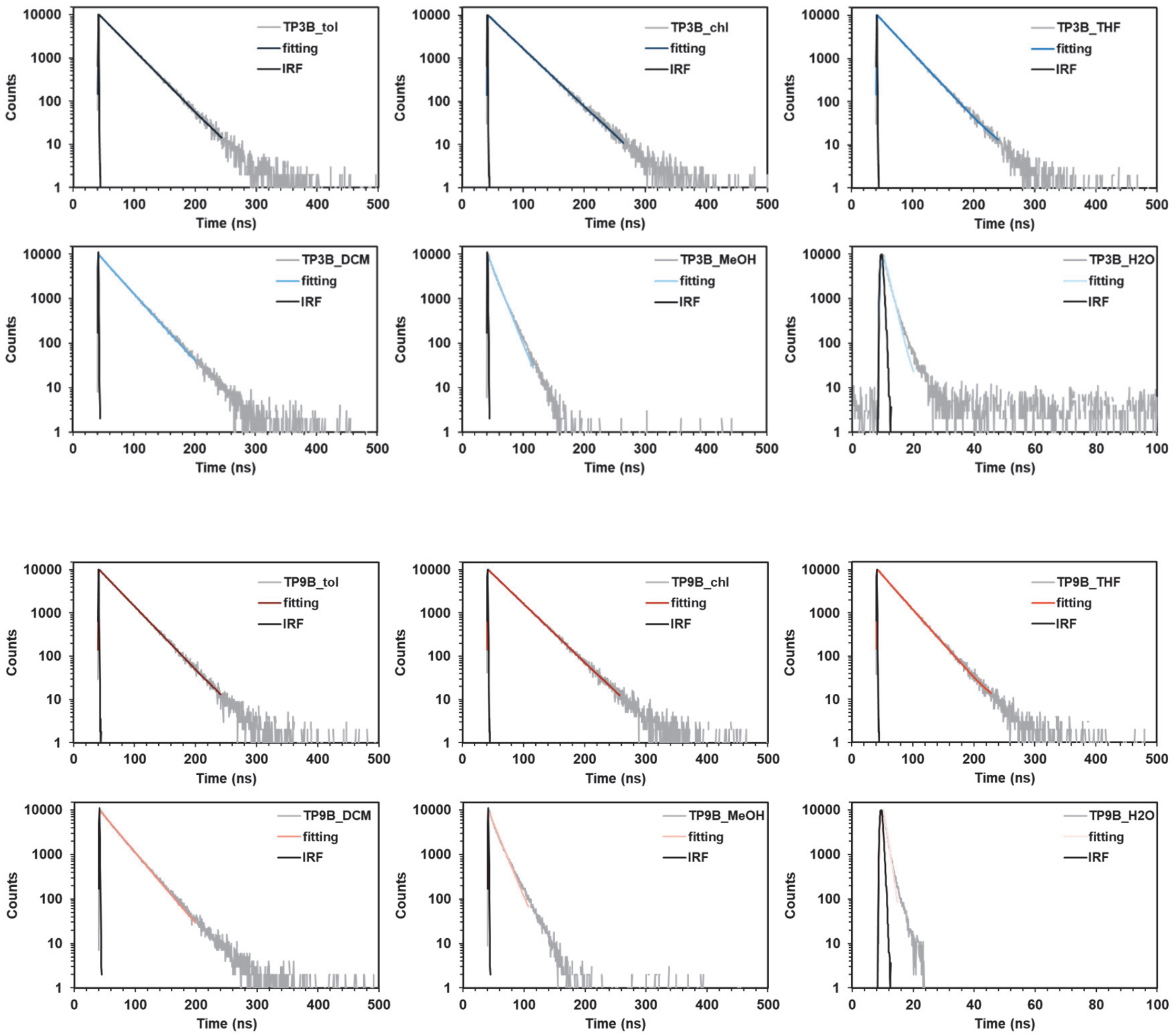

Figure S 15. PL lifetime of TTM-*tris*TP3B (TP3B) and TTM-*tris*TP9B (TP9B) in toluene (tol), chloroform (chl), tetrahydrofuran (THF), dichloromethane (DCM), methanol (MeOH), and water (H2O). The radical concentrations were prepared to be 10^{-5} M in each solvent. The black line is a reference (IRF).

Table S 2. Photophysical properties of water-soluble luminescent TTMs.

radicals	Solvent (E_T(30))	λ_{abs} (nm)	λ_{PL} (nm)	λ_{abs} - λ_{PL} (cm^{-1})	ϕ_{PL} (%)	τ (ns)	k_r (s^{-1})	k_{nr} (s^{-1})
**TTM-*tris*TP3B**	Toluene (33.9)	421/572	646	2003	47	30	1.6×10^7	1.8×10^7
	THF (37.5)	420/576	654	2071	46	29	1.6×10^7	1.9×10^7
	Chloroform (39.1)	417/558	649	2513	50	32	1.6×10^7	1.6×10^7
	Dichloromethane (40.7)	416/576	655	2094	48	29	1.7×10^7	1.8×10^7
	Methanol (55.4)	416/579	659	2097	16	13	1.3×10^7	6.7×10^7
	Water (63.1)	413/571	690	3020	1.4	1.5	9.3×10^6	6.6×10^8
	Deuterated water	414/573	689	2938	3.1	3.3	9.4×10^6	2.9×10^8
**TTM-*tris*TP9B**	Toluene (33.9)	422/560	651	2496	48	30	1.6×10^7	1.7×10^7
	THF (37.5)	420/580	659	2067	43	27	1.6×10^7	2.1×10^7
	Chloroform (39.1)	417/557	650	2569	48	32	1.5×10^7	1.6×10^7
	Dichloromethane (40.7)	416/557	661	2825	41	27	1.5×10^7	2.2×10^7
	Methanol (55.4)	417/570	665	2506	16	13	1.2×10^7	6.4×10^7
	Water (63.1)	416/564	685	3132	1.1	0.9	1.2×10^7	1.1×10^9
	Deuterated water	417/573	692	3001	3.6	2.0	1.8×10^7	4.8×10^8

DFT calculations

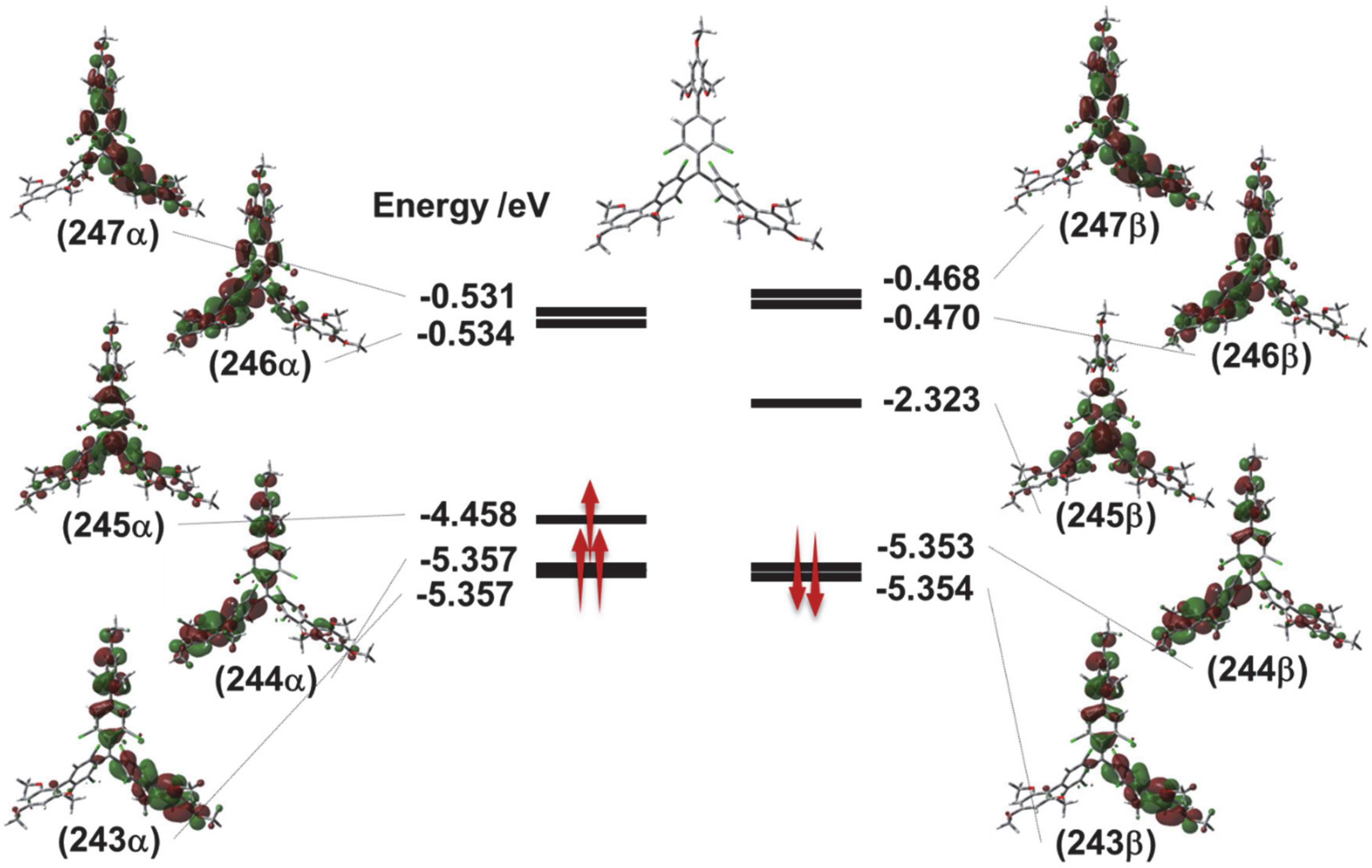

Figure S 16. Energy levels and spatial distributions of the frontier molecular orbitals of the structurally optimized methoxylated TTM obtained from DFT calculations at the UB3LYP/6-31G(d,p) level.

Table S 3. Excited states of methoxylated TTM at the structurally optimized ground state (D_0). Major configurations with the absolute value of the configuration interaction (CI) coefficient greater than 0.30000 are listed.

Excited states	Excitation energy /eV	Excitation energy /nm	Oscillator strength	Major configuration (CI Coefficients)
$D_0 \rightarrow D_1$	2.4447	507.16	0.1098	244β →245β (0.86371)
$D_0 \rightarrow D_7$	3.0020	413.00	0.2922	245α →246α (0.68722)<br>244β →245β (−0.43571)
$D_0 \rightarrow D_8$	3.0030	412.87	0.2915	245α →247α (0.68565)<br>243β →245β (−0.43665)

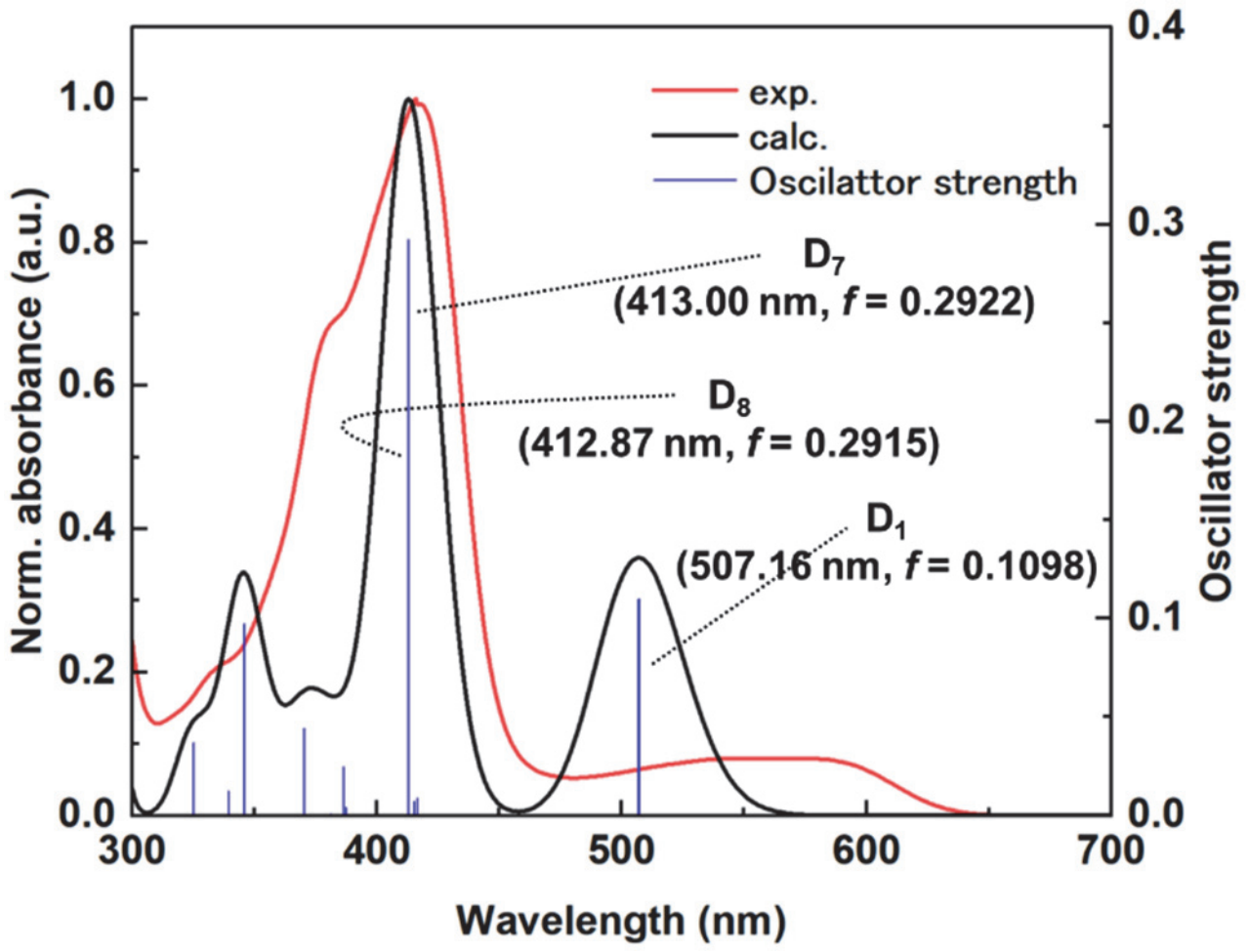

Figure S 17. Calculated (calc.) and experimental (exp.) UV-vis absorption spectra of methoxylated TTM. The calculated spectrum and oscillator strengths of each transition were obtained by the TD-DFT calculation at UPBE0/6-31+G(d,p) level. The *f* value is the calculated oscillator strength.

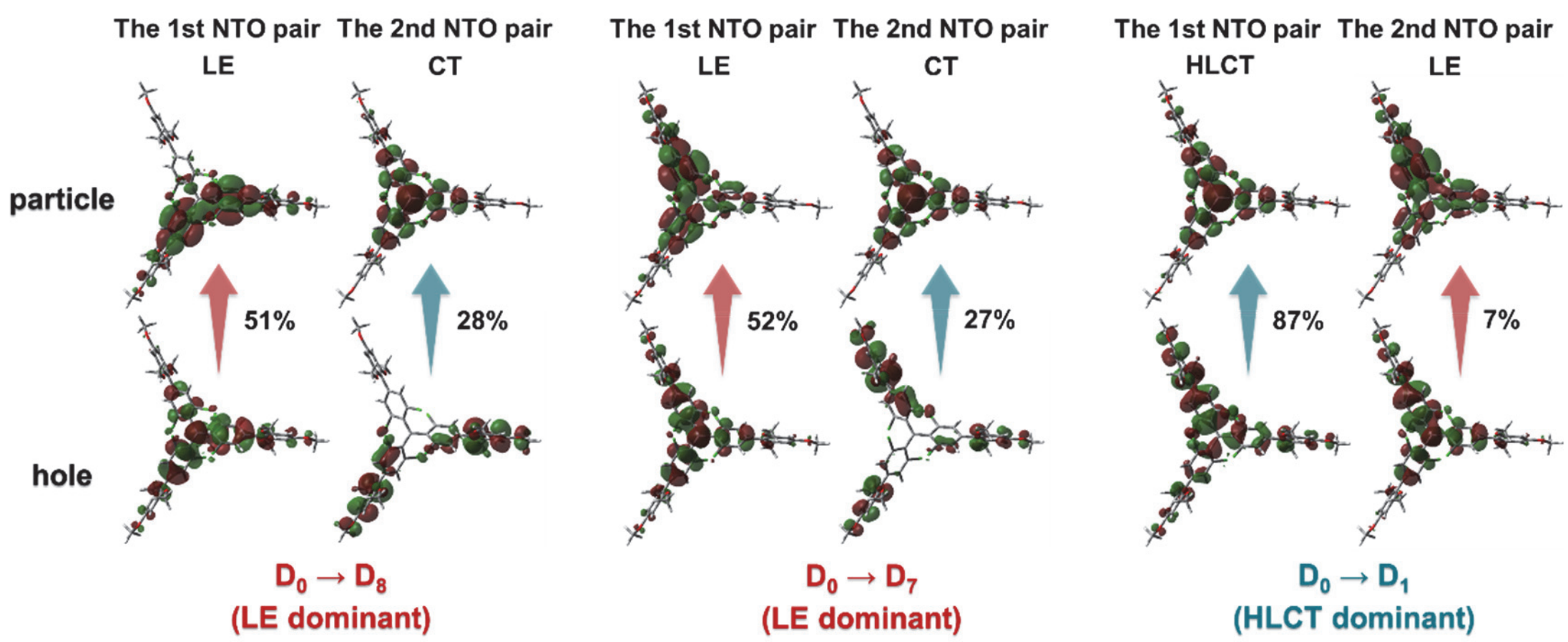

Figure S 18. The results of NTO analysis for methoxylated TTM.

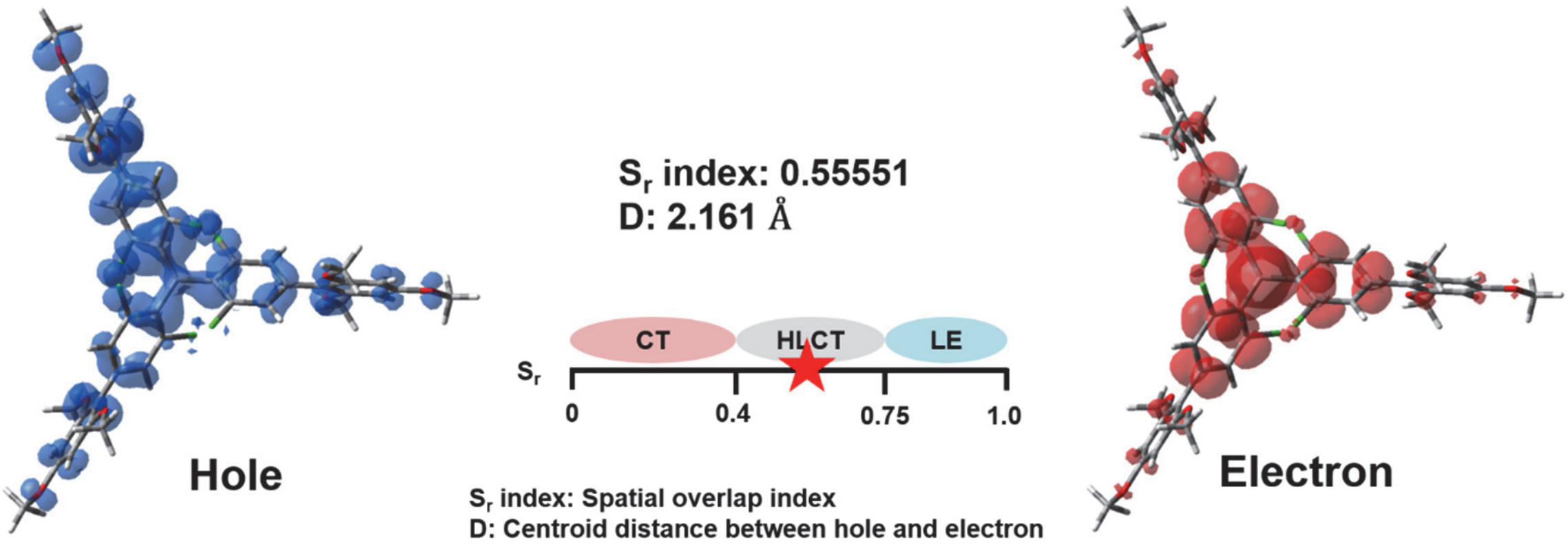

Figure S 19. The visualized hole and electron density of the methoxylated TTM radical in toluene.

Photostability

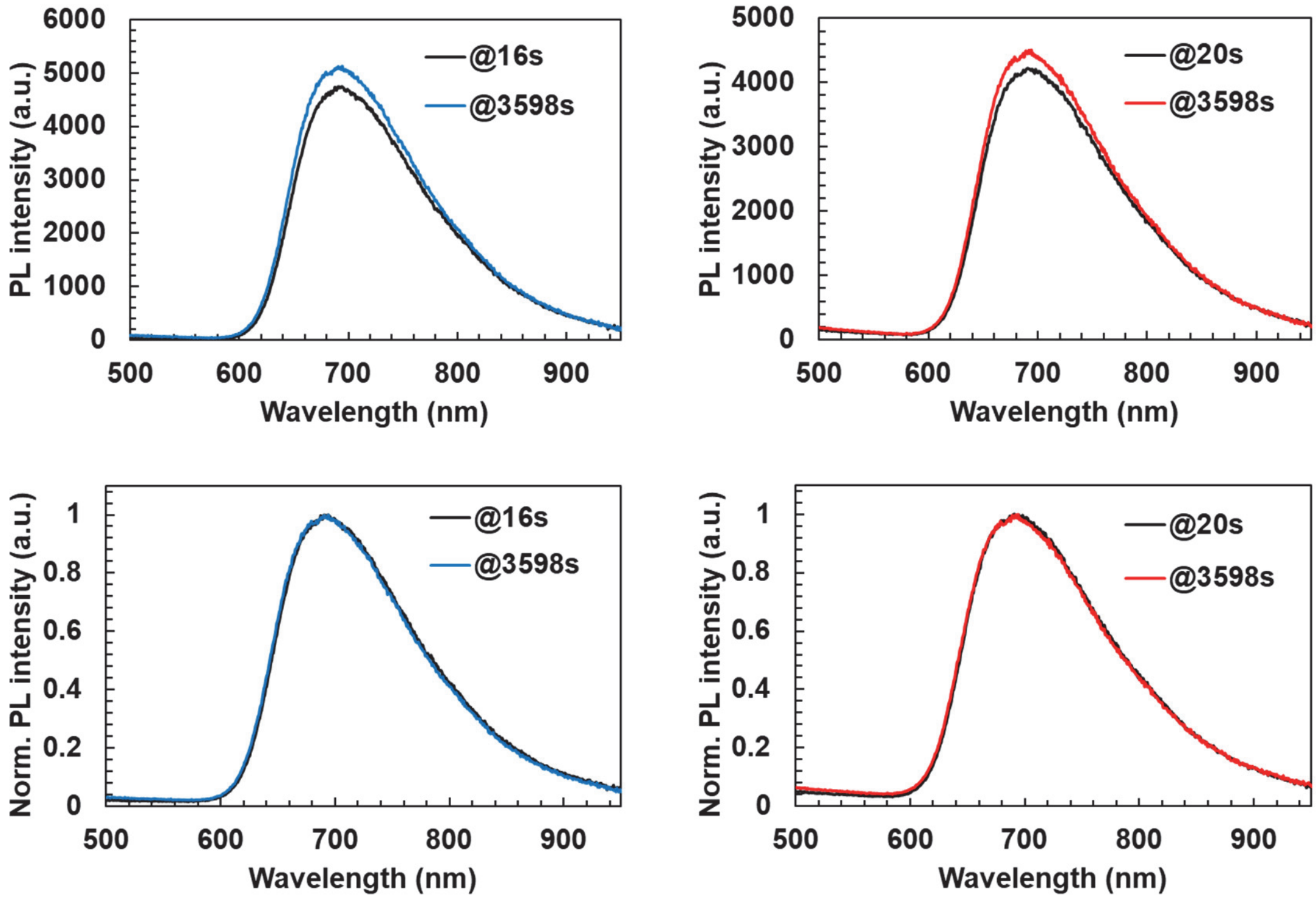

Figure S 20. PL spectra of (left) TTM-*tris*TP3B and (right) TTM-*tris*TP9B in water under continuous irradiation of a 355 nm pulsed laser. Black lines are the PL spectra at the initial stages of laser irradiation, and blue and red lines are the final stages of laser irradiation. The bottom is the normalized PL spectra.

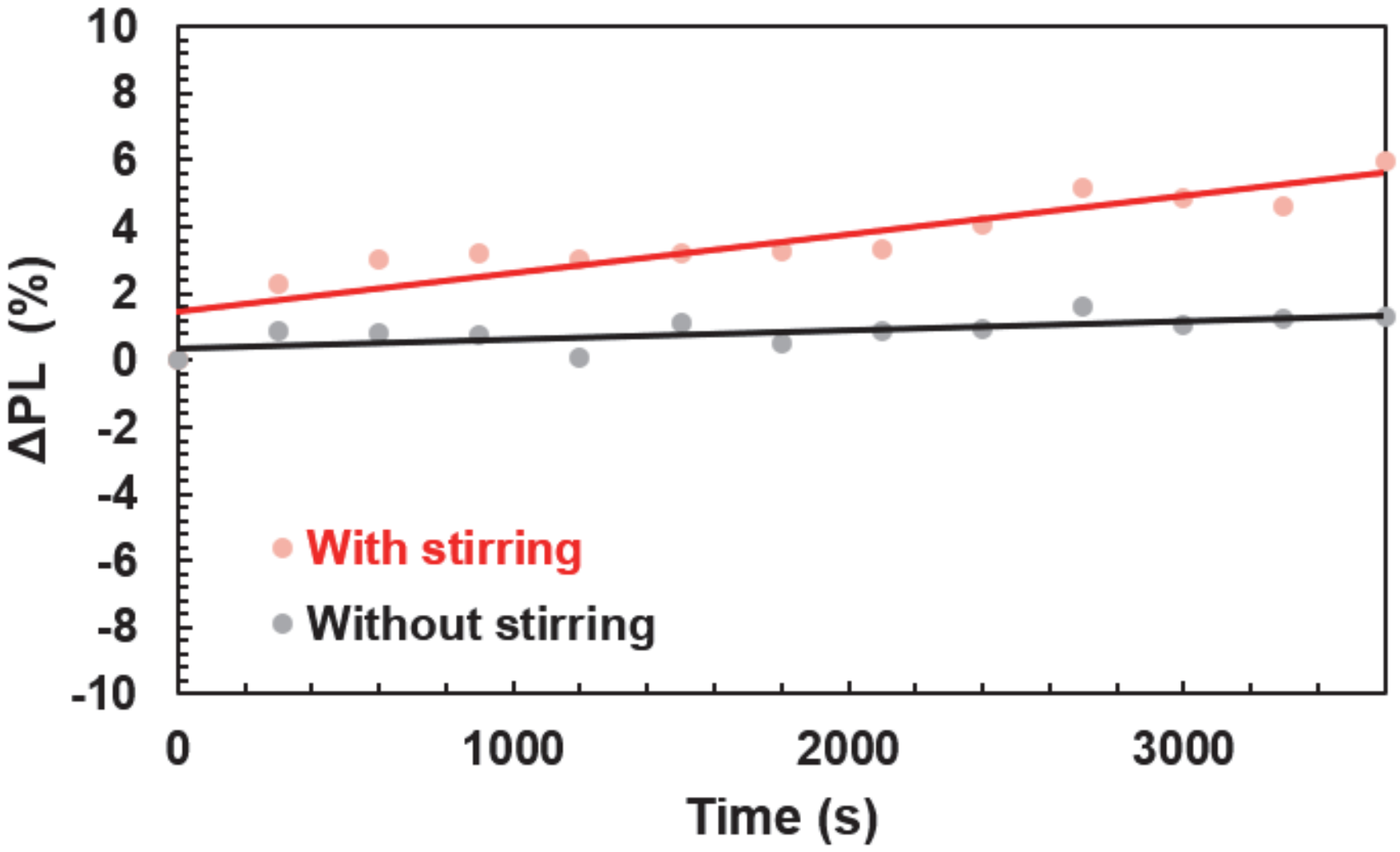

Figure S 21. The PL intensity changes of TTM-*tris*TP3B in water under intermittent laser irradiation only once every 5 min with continuous stirring or without stirring.

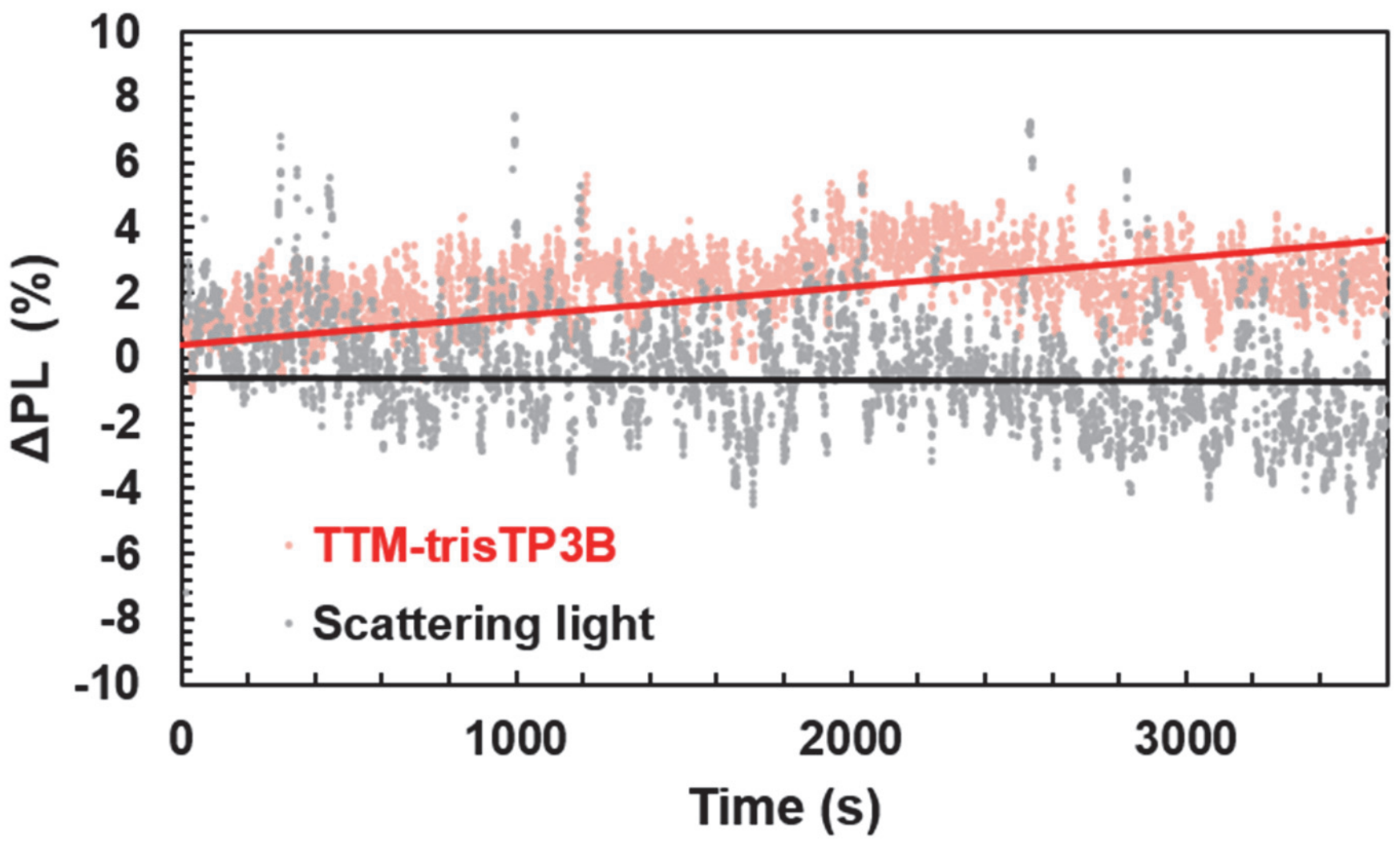

Figure S 22. The PL intensity changes of TTM-*tris*TP3B and scattering light from the excitation laser in water under continuous laser irradiation during continuous stirring.

Fluorescence imaging

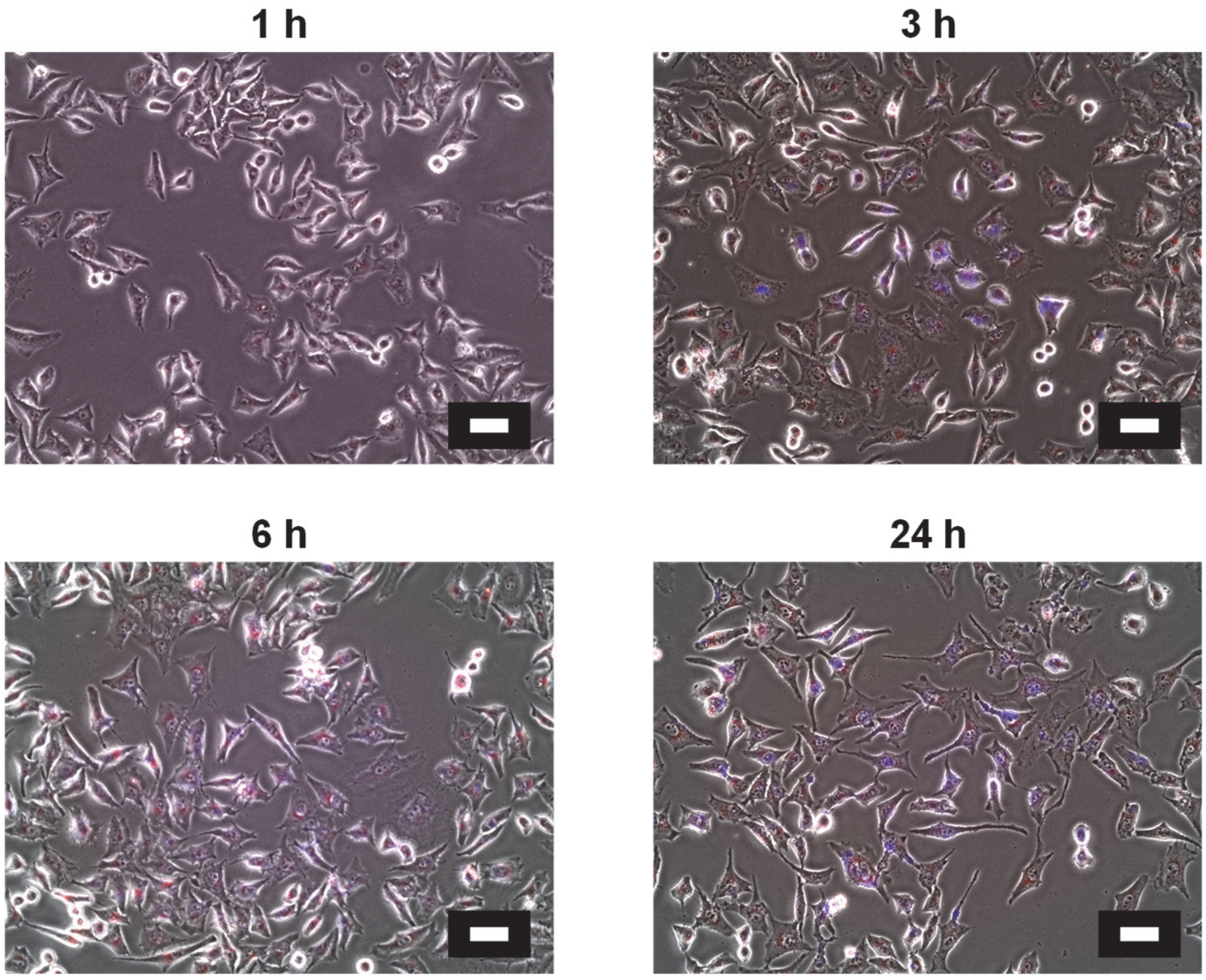

Figure S 23. Fluorescence imaging of HeLa cells incubated with 10 μM of TTM-trisTP3B for 1, 3, 6, and 24 h. Blue emissions originated from 4',6-diamidino-2-phenylindole (DAPI). Scale bars: 50 μm.

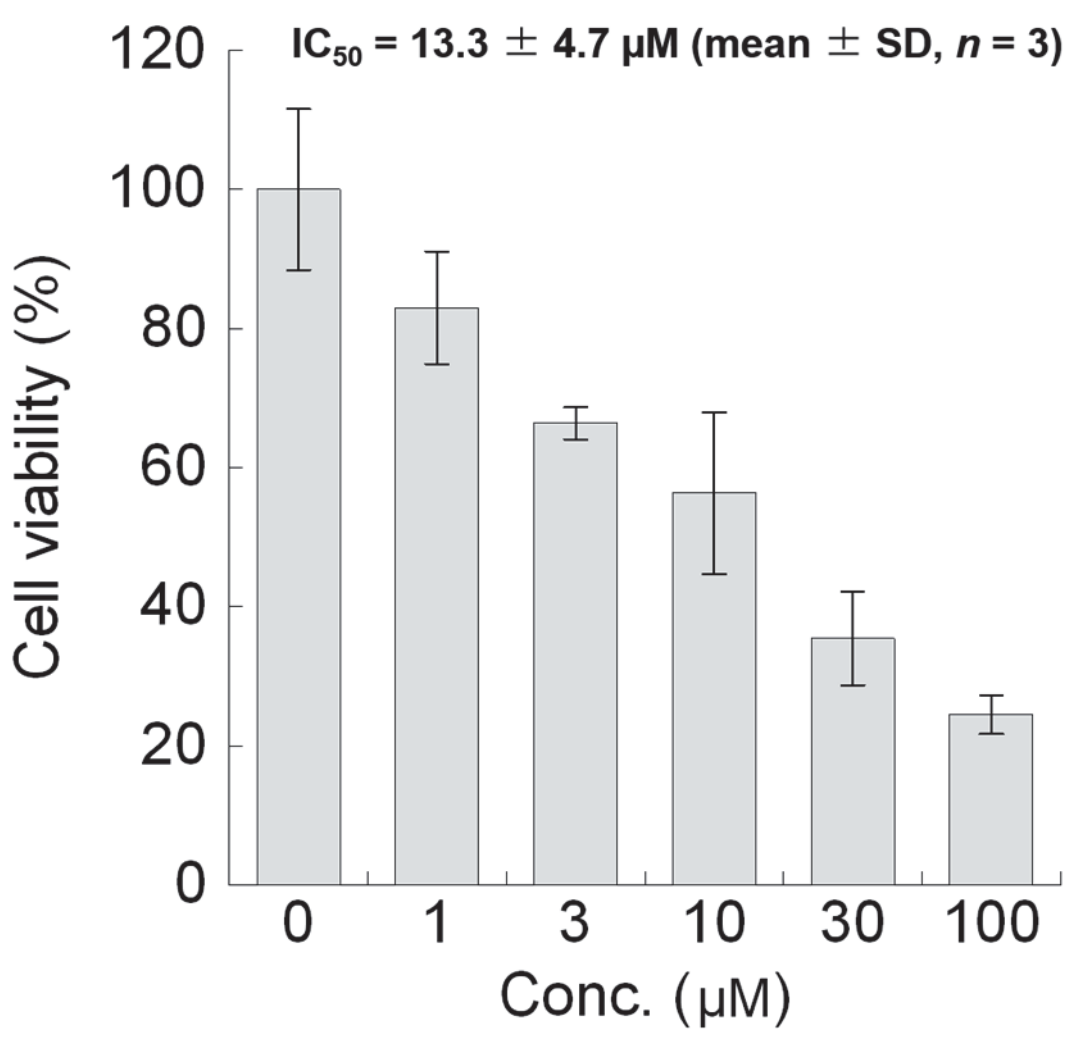

Figure S 24. Cell viability of HeLa cells treated with various concentrations of TTM-*tris*TP3B for 48 h. Data are presented as the mean ± SD (n = 3).

MRI experiments

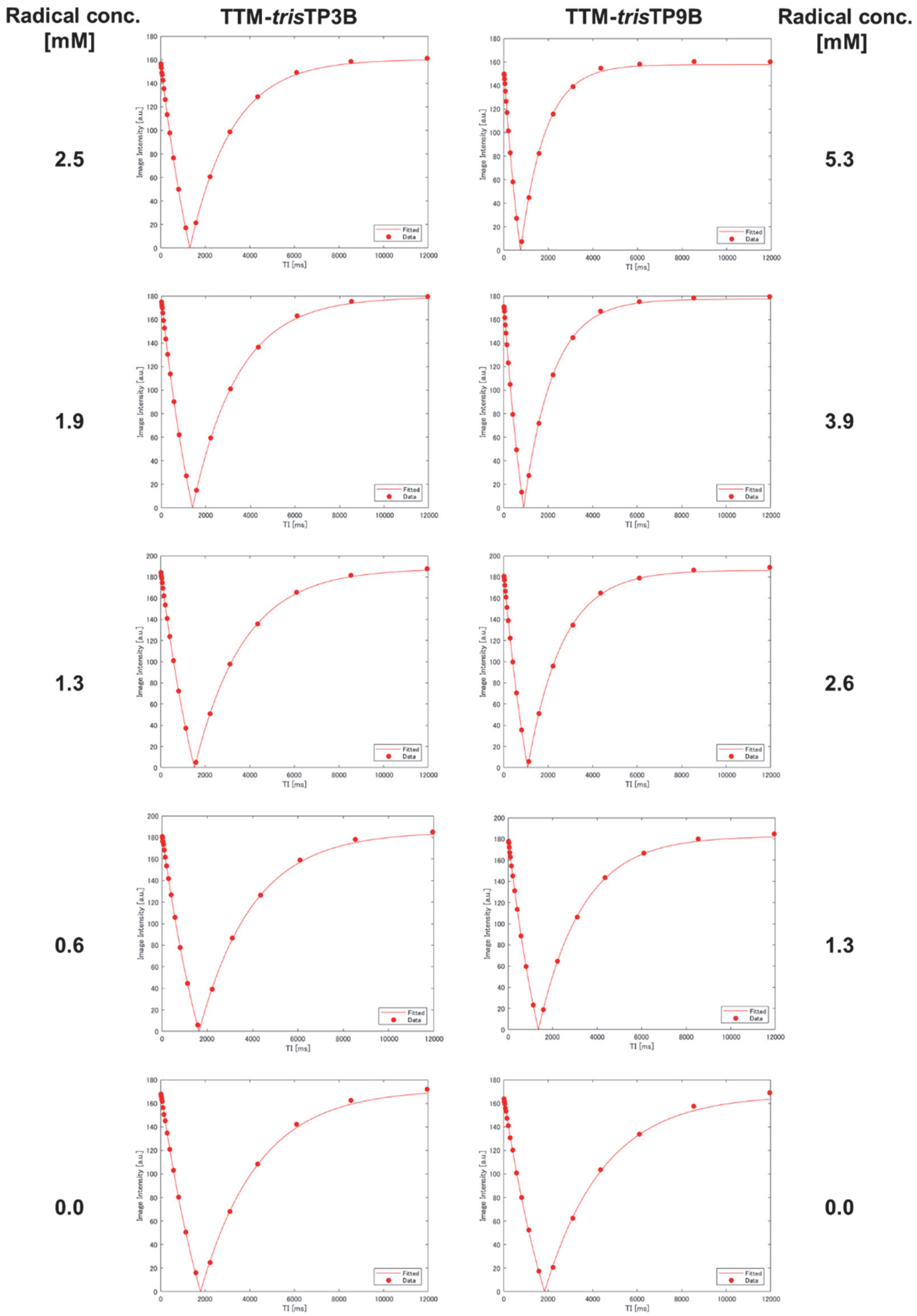

Figure S 25. Representative inversion recovery curves showing the signal intensity on T_1-weighted images as a function of inversion time (TI) for TTM-trisTP3B (left column) and TTM-trisTP9B (right column) at different radical concentrations. Each plot corresponds to a specific concentration (mM), indicated alongside the panels. Data points represent measured signal intensities, and solid lines indicate fitted curves used for T_1 estimation.

Table S 4. ***T_1*** **values of TTM-*tris*TP3B and TTM-*tris*TP9B acquired from** ***T_1*** **maps.**

radicals	concentrations (mM)	T_1 (ms)
TTM-*tris*TP3B	2.5	1897.7
	1.9	2034.6
	1.3	2197.5
	0.6	2368.8
	0.0	2588.7
TTM-*tris*TP9B	5.3	1093.6
	3.9	1300.2
	2.6	1561.9
	1.3	1967.6
	0.0	2658.1

DLS measurements

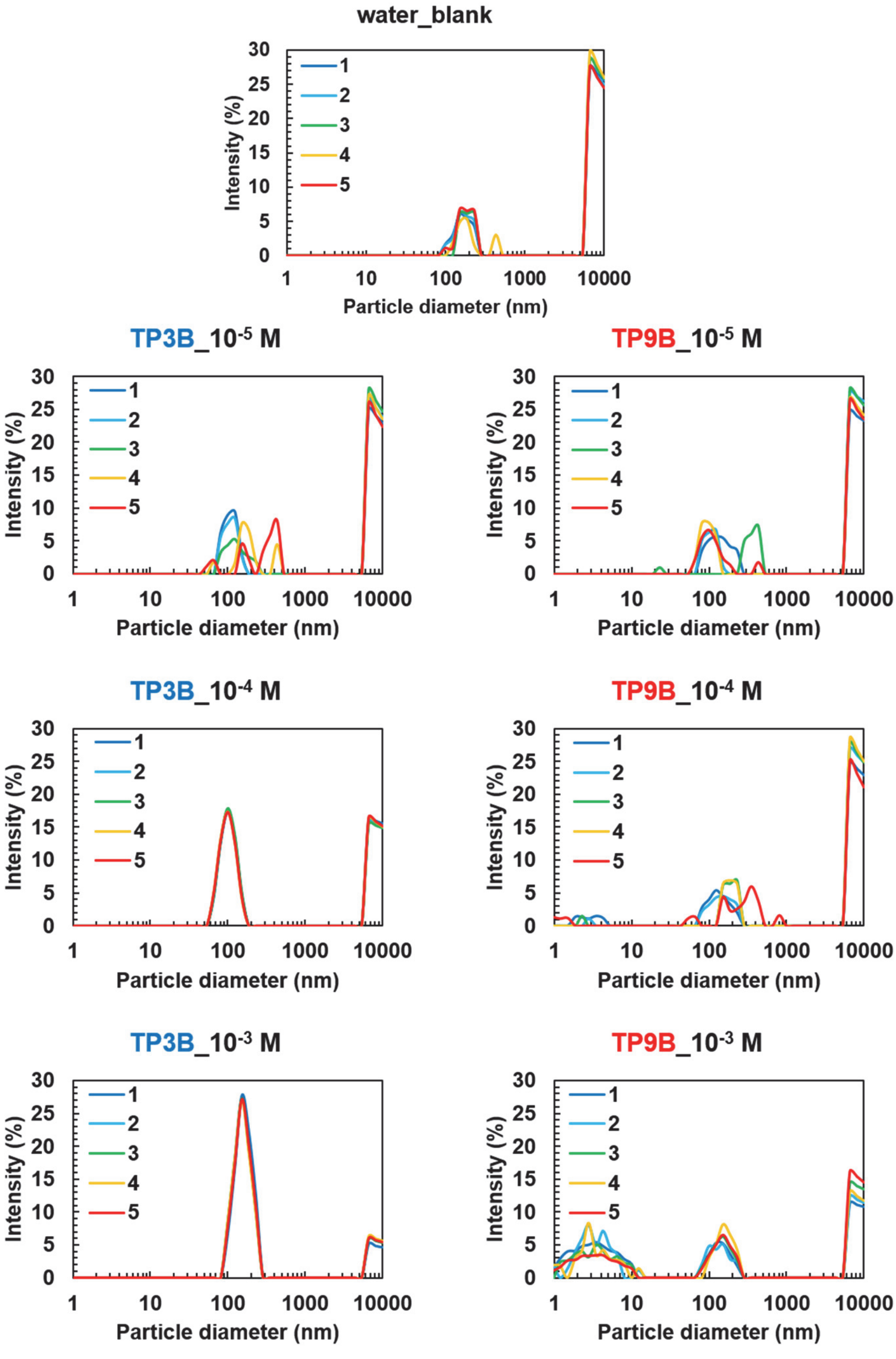

Figure S 26. The concentration-dependent particle size distributions of HTTM-*tris*TP3B and HTTM-*tris*TP9B aqueous solutions (10^{-5}, 10^{-4}, and 10^{-3} M) obtained by DLS measurement. A blank sample was prepared by filtering Milli-Q water through a 0.45 μm pore-size filter, and two unremovable peaks (a dust peak above 5000 nm and an unknown peak at around 100 nm) were observed. Radical precursor aqueous solutions were prepared to be 10^{-5}, 10^{-4}, and 10^{-3} M using the same batch of Milli-Q water as the blank sample and filtered through a 0.45 μm pore-size filter before the measurements.

Reference

1. M. J. Frisch, G. W. Trucks, H. B. Schlegel, G. E. Scuseria, M. A. Robb, J. R. Cheeseman, G. Scalmani, V. Barone, G. A. Petersson, H. Nakatsuji, X. Li, M. Caricato, A. V. Marenich, J. Bloino, B. G. Janesko, R. Gomperts, B. Mennucci, H. P. Hratchian, J. V. Ortiz, A. F. Izmaylov, J. L. Sonnenberg, D. Williams-Young, F. Ding, F. Lipparini, F. Egidi, J. Goings, B. Peng, A. Petrone, T. Henderson, D. Ranasinghe, V. G. Zakrzewski, J. Gao, N. Rega, G. Zheng, W. Liang, M. Hada, M. Ehara, K. Toyota, R. Fukuda, J. Hasegawa, M. Ishida, T. Nakajima, Y. Honda, O. Kitao, H. Nakai, T. Vreven, K. Throssell, J. A. Montgomery, Jr., J. E. Peralta, F. Ogliaro, M. J. Bearpark, J. J. Heyd, E. N. Brothers, K. N. Kudin, V. N. Staroverov, T. A. Keith, R. Kobayashi, J. Normand, K. Raghavachari, A. P. Rendell, J. C. Burant, S. S. Iyengar, J. Tomasi, M. Cossi, J. M. Millam, M. Klene, C. Adamo, R. Cammi, J. W. Ochterski, R. L. Martin, K. Morokuma, O. Farkas, J. B. Foresman, and D. J. Fox, Gaussian, Inc., Wallingford CT, 2019.
2. R. Kuwano, E. Lee, S. Won, "Economical and Readily Accessible Preparation of *o*,*o*-Disubstituted Arylboronates through Palladium-Catalyzed Borylation of Haloarenes" *Org. Lett.* **2021**, *23*, 9649.
3. K. Walter, D. P. Hoch, L. Hertweck, K. Balasubramanian, J. Geisler, M. Röllig, T. J. Neubert, H. G. Börner, "Unlocking the Essence of Lignin: High-Performance Adhesives That Bond via Thiol-Catechol Connectivities and Debond on Electrochemical Command" *Adv. Mater*. **2025**, *37*, e10463.
4. G. M. Pitch, L. N. Matsushima, Y. Kraemer, E. A. Dailing, A. L. Ayzner, "Energy Transfer in Aqueous Light Harvesting Antennae Based on Brush-like Inter-Conjugated Polyelectrolyte Complexes" *Macromolecules* **2022**, *55*, 10302.
